# Melanoma: Pathogenesis and Targeted Therapy

**DOI:** 10.1002/mco2.70566

**Published:** 2026-01-04

**Authors:** Yang Fu, Jie Liu, Zeming Mo, Bin Wang, Yaotiao Deng, Yu Jiang

**Affiliations:** ^1^ Department of Medical Oncology Cancer Center West China Hospital Sichuan University Chengdu China

**Keywords:** BRAF, immunotherapy, melanoma, NRAS, targeted therapy

## Abstract

Melanoma is the most aggressive skin malignant tumor, typically exhibiting a high mutation burden and potentially harboring mutations in NRAS, BRAF, or NF1. To enhance survival rates, these driver alterations can achieve significant antitumor activity through targeted therapy. In the past decade, BRAF inhibitors combined with MEK inhibitors significantly improved the prognosis of BRAF mutation melanoma. Nevertheless, researchers have attempted various strategies to block the NRAS signaling pathway, NRAS mutation in melanoma is still considered to be untargetable. In recent years, MEK inhibitors like binimetinib and tunlametinib have displayed the efficacy for NRAS^mut^ melanoma, with tunlametinib being the first and only approved MEK inhibitor for advanced NRAS^mut^ melanoma. On the other hand, immune checkpoint inhibitors including PD‐1/PD‐L1 inhibitors and cytotoxic T‐lymphocyte antigen 4 （CTLA‐4） inhibitors changed the treatment landscape of advanced melanoma. In this review, we have summarized the current knowledge of molecular pathogenesis and classification of melanoma. Subsequently, we explored current and potential treatment approaches for melanoma, primarily encompassing BRAF inhibitors, MEK inhibitors, and immunotherapy, with a particular focus on their clinical relevance of development. Finally, the challenges in the treatment of melanoma, particularly in immunotherapy and targeted therapy, are summarized and discussed.

## Graphical abstract

The treatment landscape for advanced melanoma has evolved significantly. BRAF–MEK inhibitor combinations are standard for BRAF‐mutant melanoma, whereas targeting NRAS mutations is more challenging, with MEK inhibitors like tunlametinib emerging as an option. Alongside these developments, immune checkpoint inhibitors have become a cornerstone therapy. This review summarizes the molecular classification of melanoma, synthesizes current therapeutic approaches including targeted and immunotherapy, and highlights persistent clinical challenges.

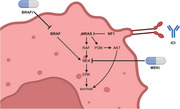

## 1 Introduction

Melanoma is a highly aggressive malignant tumor, which is derived from normal melanocytes. Over 280,000 cases (1.6%) of all newly diagnosed primary malignant cancers are cutaneous melanoma, and there are approximately 60,000 cancer deaths (0.6% of cancer deaths) due to cutaneous melanoma in the world [1, 2]. The incidence of melanoma varies across different regions, with Southeast Asia reporting a lower incidence rate of 0.2 per 100,000 person‐years compared with New Zealand (35.8 per 100,000 person‐years), Australia (34.8 per 100,000 person‐years), the EU (13.8 per 100,000 person‐years), and the United States (7.7 per 100,000 person‐years) [3]. In recent decades, 5‐year survival rate of nonmetastatic melanoma has significantly improved due to the improvement of diagnosis, screening approaches, surgery, and novel drugs. However, the prognosis of distant metastasis melanoma is still low, with a 5‐year survival rate of about 27% [4, 5].

Caucasians in Western countries mainly suffer from cutaneous melanoma, and ultraviolet exposure was recognized as a crucial factor in its etiology [6]. Nevertheless, non‐Caucasians populations in Asia and Africa predominantly develop acral melanomas, which accounts for nearly 60% of all diagnosed melanoma cases in these areas [6, 7]. In addition, acral melanomas account for around 45% of melanoma cases in Chinese population [8]. The etiology of acral melanoma in Asia is still uncertain, although risk factors such as injury and mechanical stress of the feet/hands and chronic inflammation have been considered [7, 9, 10]. Mucosal melanoma is a rare subtype (1–2%) of melanomas in Caucasians population [11, 12], while in Asian countries, mucosal melanoma is another predominant melanoma subtype, accounting for 20–30% [13, 14, 15]. The incidence rate of uveal melanoma also varies among racial groups and is more common among non‐Hispanic white individuals (6.02 per 100,000 person‐years) than Hispanic (1.67 per 100,000 person‐years), Asian (0.38 per 100,000 person‐years), or Black individuals (0.31 per 100,000 person‐years) [16].

BRAF and NRAS mutations are the most common in melanoma. In the last two decades, targeted therapy has significantly improved the prognosis of melanoma with BRAF mutation, with vemurafenib and dabrafenib being approved by United States Food and Drug Administration (US FDA) [17, 18]. Subsequent studies have shown that combination therapy of BRAF and MEK inhibitors provides better outcomes than single agent of BRAF inhibitor for BRAF^mut^ melanoma, becoming the current standard scheme [19, 20]. Compared with BRAF mutant melanoma, only tunlametinib has been approved and available for clinical in NRAS mutant melanoma. Several efforts have been made in past decades to identify druggable therapy in NRAS signaling pathway including NRAS related posttranslational modifications, direct NRAS targeting, and mitogen‐activated protein kinase (MAPK) pathway inhibitors.

Immunotherapy, such as programmed cell death 1 (PD‐1)/programmed cell death ligand 1 (PD‐L1)/cytotoxic T‐lymphocyte antigen 4 (CTLA‐4) inhibitors, is another parallel approach for the treatment of melanoma. Several Phase II/III studies tested the efficacy of immune checkpoint inhibitors (ICIs) combined with BRAF/MEK inhibitors for BRAF^mut^ melanoma and the results were desirable [21, 22, 23, 24].

This review aims to provide a comprehensive overview of the signaling pathways involved in BRAF^mut^ and NRAS^mut^ melanoma and the current treatment strategies, which include targeted therapy and immunotherapy. We also provide an updated overview of ongoing clinical trials and discuss the future challenges of these therapies. This comprehensive overview of BRAF^mut^ and NRAS^mut^ melanoma, aiming to explore the optimal treatment for these melanoma subtypes and guide the development of more effective therapies based on their signaling pathways. All these are key to improving the survival of patients with BRAF/NRAS mutation melanoma.

## 2 Molecular Pathogenesis and Classification of Melanoma

### 2.1 Melanoma Subtypes and Diagnosis

According to the 2018 WHO classification of skin tumors, melanomas were divided into nine subtypes: superficial spreading melanoma, lentigo maligna melanoma, desmoplastic melanoma, Spitz melanomas, acral melanomas, mucosal melanomas, melanomas arising in congenital nevi, melanomas arising in blue nevi, and uveal melanoma [25]. Traditionally, melanoma can also be classified based on anatomical location into cutaneous melanomas (skin melanomas without chronic sun‐induced damage, skin melanomas with chronic sun induced damage), mucosal melanomas, and acral melanomas [26]. Based on the histopathological characteristics of melanoma, melanoma can be divided into four main types: superficial spreading melanoma, nodular melanoma, lentigo maligna melanoma, and acral lentiginous melanoma [3].

Histopathology is the gold standard for the diagnosis of melanoma [27]. The differentiation of melanocytic lesions relies on specific markers unique to melanocytes, which are associated with melanosomes not expressed in other cell lines. The maturation of melanosomes is a four‐stage process. During this process, the lipid membrane of the organelle starts to incorporate the melanosome‐specific protein PMEL17/gp100, followed by the synthesis of tyrosinase and dopachrome tautomerase, and ultimately, the organelle contains MART‐1/Melan‐A [28]. Based on this, melanocytes can be identified through immunohistochemical detection of melanosomal proteins PMEL17/gp100, MART‐1, or tyrosinase. Since the transcription factors microphthalmia‐associated transcription factor (MITF) and SOX10 (SRY‐related HMG‐box gene 10) in melanocytes govern gene transcription, immunohistochemical detection of these factors can also serve as specific markers for melanocytes [29]. S100B (S100 calcium‐binding protein), a widely utilized yet less specific marker for melanocyte proteins, is also expressed by nerve cells [30]. Meanwhile, the challenge in diagnosis is often to exclude other benign lesions. Immunohistochemical staining for the nucleoprotein Ki67 is not appropriate for making this distinction, as moles, particularly those subjected to mechanical trauma, may contain proliferating nevus cells [31]. A viable approach involves utilizing immunohistochemical markers for malignant tumors: currently, two such markers, p16 and PRAME, have undergone evaluation and validation [32]. The mere absence of p16 protein may not suffice to accurately differentiate between benign and malignant lesions; however, combining its detection with Ki67 and PMEL/gp100 might better fulfill diagnostic requirements [28, 31].

### 2.2 Key Driver Mutations and Signaling Pathways

#### 2.2.1 BRAF/NRAS Mutation

BRAF gene encodes cytoplasmic serine/threonine kinase regulated by Ras and implicated in the MAPK signal transduction [33]. In 2002, a genome‐wide screening discovered that 66% of melanoma had BRAF somatic missense mutations, which were more frequent than other types of solid tumors. About 50% of melanoma detect that valine replaces glutamate at codon 600 (V600E), leading to activation of BRAF protein kinase and downstream MAPK pathway [33]. Other mutations including V600K, V600D, and V600R accounted for 12–15, 5, and 1–1.8% of BRAF mutations, respectively [34, 35].

Neuroblastoma rat sarcoma (NRAS) is an oncogene that belongs to RAS family (including NRAS, KRAS, and HRAS). These oncogenes encode small GTP‐binding proteins that respond to upstream RTK activation to promote the activity of downstream RAF signaling [36, 37]. Somatic mutations of NRAS, KRAS, and HRAS occur in 20–25, 2–3, and 2% of melanomas respectively [35, 38, 39, 40, 41]. Single point mutation of RAS cause one‐third of all human cancers. The majority of NRAS mutations involving codon 61 (60–82%) and 14–35% involving codon 12 or codon 13. The most common NRAS mutations are Q61R (38%) and Q61K (31%) [37, 39]. NRAS mutation status is an independent predictor of shorter overall survival (OS) in metastatic melanoma compared with BRAF mutation or BRAF/NRAS wild‐type melanoma [42, 43].

#### 2.2.2 NRAS/BRAF Mutation in Different Subtypes of Melanoma

The frequency of NRAS mutations varies among different subtypes and races of melanoma [44]. About 25% cutaneous melanoma in the Caucasians population carry NRAS mutations, compared with only about 2.5% in the Asian population [35, 40]. In Caucasians population, the rate of acral melanoma with NRAS mutations is about 15–20% [45, 46]. While in Asian population, the frequency of NRAS mutations in acral melanoma seems to be lower. In a study from Korean, NRAS alterations were found in only 4.3% in 47 acral melanoma patients [47]. And NRAS alterations were present in 12.0% of 83 Chinese patients [48]. Another Chinese study included 311 samples of acral melanoma, and only one sample (1.4%) was observed NRAS mutation in 70 assessable samples [49]. Additionally, the frequency of NRAS in mucosal melanoma is considerably lower than that in cutaneous melanoma. Dumaz et al. summarized 36 publications, and the results showed that the frequency of NRAS mutations was 12% (179 out of 1454) in mucosal melanoma [50]. In a meta‐analysis, NRAS mutations were observed in 8% of 9223 mucosal melanoma [51]. Another multicenter retrospective research showed that 18% (37 out of 198) of mucosal melanoma harbored NRAS mutations [52]. BRAF mutations frequency has little difference among races, BRAF mutations in cutaneous melanoma account for approximately 50% in Caucasians and Asians, and BRAF mutations in acral melanoma account for about 15% in Caucasians and Asians [35, 40]. Uveal melanoma is an uncommon and special subtype of melanoma with a unique mutation landscape, lacking NRAS, BRAF, and NF1 mutation [53].

#### 2.2.3 Key Oncogenic Signaling Pathways

**FIGURE 1:**
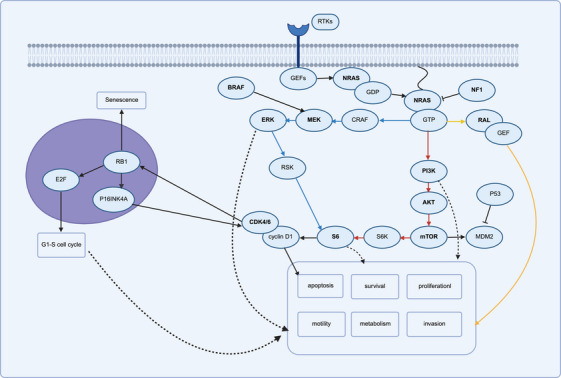
Schematic representation of NRAS^mut^/BRAF^mut^/NF1^mut^ signaling pathways including MAPK pathway, PI3K/AKT pathway, and RAL pathway. Blue line: MAPK pathway; red line: PI3K/AKT pathway; yellow line: RAL pathway; dashed line: indirect effect.

The RAS proteins, encoded by the NRAS gene, mainly reside on the cytoplasmic side of the plasma membrane but can also be directed to other membrane sites by adding specific lipid moieties to their carboxyl termini [54, 55, 56]. Activation of receptor tyrosine kinases, such as epidermal growth factor receptor (EGFR), lead to the recruitment of RAS guanine nucleotide exchange factors (GEFs) like son‐of‐sevenless (SOS) to the plasma membrane. These GEFs facilitate the exchange of inactive GDP‐bound state for active GTP‐bound state on RAS [57, 58]. GTP‐bound RAS, through its RAS‐binding domain (RBD), binds a large number of effectors (proteins), including RAF kinases, phosphoinositide 3‐kinase (PI3K) and RAL GEFs, which transmit a series of pro‐proliferative and other signals within the cell. The binding of GTPase activating proteins (GAPs) to GTP‐bound RAS accelerates the GTPase activity of RAS, returning it to an inactive GTP‐bound state and ultimately terminating signal transduction. RAS gene mutations are typically characterized by single base missense mutations, with residues G12, G13, or Q61 accounting for approximately 99% of the mutations [59]. These single base missense mutations result in a loss of GAPs activity, causing RAS to remain in a GTP‐bound and active state, continuously activating multiple downstream pathways that have been demonstrated to contribute to carcinogenesis [56, 58, 60, 61]. The classic RAS‐mediated downstream pathways are the MAPK pathway, the PI3K/protein kinase B (AKT) cascade pathway, and RAS‐like (RAL) pathway (Figure 1). In NRAS mutation melanoma cells, activation of the MAPK is achieved through the activation of CRAF, rather than BRAF in normal melanocytes [62, 63, 64]. For BRAF mutated melanoma cells, abnormal activation of RAF protein kinase causes MEK phosphorylation and activates downstream pathways, such as ERK1 and ERK2 [29]. Previous studies have demonstrated that MAPK pathway play a dominate role in NRAS/BRAF mutant melanoma compared with the PI3K pathway [63, 65]. The effector p70 ribosomal S6 kinase (P70S6K, known as S6K1) in the PI3K downstream pathway and the effector p90 ribosomal S6 kinase (RSK) in the MAPK downstream pathway can both phosphorylate ribosomal protein S6, leading to its activation [66, 67]. In NRAS‐mutated melanoma, S6 phosphorylation mainly depends on RSK rather than S6K1, and dysregulation of phosphorylation S6 leads to upregulation of cyclin D1 (CCND1) and inactivation of the tumor suppressor p16 [INK4A^68^]. CCND1 and the catalytic CDK4/6 form active heterodimers that phosphorylate target proteins, playing crucial roles in the G1–S transition of the cell cycle and deactivation (phosphorylate) of the retinoblastoma protein (RB1). Interestingly, the tumor suppressor p16^INK4A^ can negatively regulate the deactivation of RB1 by CDK4/6, and the inactivation of CDK4/6 relieves RB1‐mediated suppression of p16 [INK4A^69^]. Moreover, activation of RAS stimulates PI3K, which in turn recruits and activates AKT kinase. Once activated, AKT modulates the mammalian target of rapamycin (mTOR) pathway and its downstream target S6K. This pathway ultimately facilitates cell cycle progression by upregulating the translation of CCND1 [56, 70]. In the RAL pathway, the signal transduction between RAS and RAL is mediated by RAL‐GEF, which promote the release of GDP from the RAL protein and its subsequent binding to GTP, similar to the effect of SOS [71, 72, 73]. Activation of RAL‐GEFs promotes the proliferation, anchorage‐dependent growth, and angiogenesis of NRAS^mut^ melanoma [73, 74, 75].

### 2.3 Molecular Subtypes and Their Clinical Relevance

The age at diagnosis of NRAS^mut^ melanoma is typically around 60–70 years, which is higher than melanoma with BRAF mutations or NRAS/BRAF wild‐type [76, 77, 78]. The proportion of male patients is slightly higher than that of females, with a ratio of about 1:0.8–0.9 [76, 77, 78]. NRAS^mut^ melanoma is more common in the trunk and extremity among Caucasians [76, 78, 79], while in Japanese population, it is more frequently present on the face, palm, and sole [77]. When diagnosed, patients with NRAS or BRAF mutations tend to have a higher AJCC stage, particularly Stage III, compared with wild‐type melanoma [79]. As the disease progresses, melanoma is prone to brain metastases, and NRAS^mut^ melanomas were identified in about 20% of melanoma brain metastases [80, 81, 82, 83].

NRAS mutation melanoma exhibits a dome‐shaped silhouette that is generally symmetrical [84]. It is associated with a higher level of invasion based on Clark level compared with NRAS wild‐type, and it has a trend of lower frequent of ulceration compared with BRAF mutation [76, 79, 84]. Additionally, NRAS mutation melanoma tends to have a thicker tumor (median thickness of about 3.7 mm) based on Breslow depth compared with BRAF mutation or NRAS/BRAF wild‐type [43, 79]. Under the microscope, NRAS mutation melanoma displayed a higher rate of mitosis (count of >1⁄mm^2^) compared with BRAF mutations and wild‐type melanoma [43, 84]. The cells of NRAS^mut^ melanoma present with round nuclei, conspicuous nucleoli, and abundant cytoplasm [84]. On the other hand, BRAF mutation melanoma shows distinct morphological features such as increased upward migration, sharper boundaries with surrounding skin, and larger, rounder, and more pigmented tumor cells [78]. Compared with BRAF^mut^ melanoma, NRAS^mut^ melanoma shows less pronounced infiltration of lymphocytes [76, 84].

## 3 Targeted Therapy for Melanoma: From Monotherapy to Rational Combinations

### 3.1 Targeting KIT‐Mutant Melanoma

C‐KIT is a Type III transmembrane RTK directly responsible for binding to growth factors and activating the MAPK and PI3K–AKT pathways [85, 86]. What is more, C‐KIT mutations and amplifications are detected in about 2% of all melanomas, and 10–20% of acral melanomas and mucosal melanomas [87]. C‐KIT inhibitors such as imatinib and nilotinib have been approved for gastrointestinal stromal tumors and have shown the efficacy of advanced melanomas with C‐KIT alterations in several clinical trials. In 2010, Guo et al. first conducted a Phase II trial of imatinib in patients with metastatic melanoma with c‐Kit alterations, and the results revealed a promising effectiveness, with an objective response rate (ORR) of 23.3% [88]. Another Phase II study of imatinib observed an ORR of 16%, a median progression‐free survival (mPFS) of 12 weeks, and a mOS of 46.3 weeks in C‐KIT alterations melanoma patients [89]. Nilotinib has a similar historical data of imatinib, with an ORR of 26.2% in the TEAM trial (NCT01028222) [90].

### 3.2 Targeting BRAF‐Mutant Melanoma

#### 3.2.1 BRAF Inhibitors of Monotherapy

BRAF inhibitors such as vemurafenib (PLX4032) and dabrafenib have demonstrated remarkable clinical activity in melanoma patients with BRAFV600 mutations [91, 92, 93]. In the Phase III trial (BRIM3), vemurafenib showed efficacy in BRAFV600 mutations patients with a mOS and mPFS of 13.6 and 5.3 months, compared with dacarbazine group with 9.7 and 1.6 months, respectively [92]. Additionally, dabrafenib showed efficacy in the BREAK3 Phase III trial. Dabrafenib group had a mOS and mPFS of 20 and 6.9 months, whereas chemotherapy group had 15.6 and 2.7 months, respectively [91]. Based on these results, the US FDA approved dabrafenib as a standard first‐line treatment for unresectable/metastatic melanoma patients with BRAFV600 mutation.

#### 3.2.2 BRAF Inhibitor Combined With MEK Inhibitors

Acquired resistance to single agent of BRAF inhibitors frequently develops through reactivation of the MAPK pathway [94, 95]. Also, BRAF inhibitors result in the development of secondary keratoacanthomas and cutaneous squamous cell carcinomas, originating from a paradoxical activation of the MAPK pathway in tumor cells [96, 97, 98]. In several clinical studies, cotargeting of BRAF together with MEK significantly delays secondary resistance, with a longer mPFS than with BRAF inhibitor monotherapy, as well as prevents formation of secondary skin tumors [19, 99, 100].

In 2014, the combination of dabrafenib and trametinib was the first combination approved for the treatment of advanced melanoma with BRAF mutation in the United States. In the Phase III COMBI‐v study, the mPFS was 11.4 months in the combination group and 7.3 months in the vemurafenib group (*p* < 0.001). The ORR was 64% for the combination group and 51% for vemurafenib group (*p* < 0.001) [101]. Another Phase III study (COMBI‐d), the combination group also showed higher ORR than dabrafenib group (69 vs. 53%) [102]. The Phase III coBRIM study showed an improvement in the ORR (68 vs. 45%; *p* < 0.001), in mPFS (9.9 vs. 6.2 months; *p* < 0.001), and in mOS (22.3 vs. 17.4 months; *p* = 0.005) when cobimetinib was added to vemurafenib [100, 103]. The combination therapy had a nonsignificantly higher incidence of adverse events (grade ≥ 3) compared with vemurafenib group, and there was no significant difference in the rate of drug discontinuation. Encorafenib (LGX818) is an ATP‐competitive BRAF inhibitor that can inhibit the MAPK pathway in tumor cells expressing various forms of BRAF kinase mutations, such as V600E, V600D, and V600K mutations. Encorafenib has more than 10 times longer dissociation half‐life than dabrafenib or vemurafenib; thus, it enables sustained target inhibition [104]. Subsequently, the third combination of BRAF inhibitor (encorafenib) plus MEK inhibitor (binimetinib) was tested in the three‐arm Phase III COLUMBUS study. In this trial, 577 advanced melanoma patients carrying the BRAFV600 mutation were randomized to encorafenib plus binimetinib (*n* = 192), encorafenib (*n* = 194), or vemurafenib (*n* = 191) monotherapy. The results observed the combination group had the highest ORR with 63 versus 51 versus 40% [105]. The mOS was 33.6 months with encorafenib plus binimetinib and 16.9 months with vemurafenib (*p* < 0.0001) [106].

### 3.3 Targeting NRAS‐Mutant Melanoma: A Therapeutic Challenge

#### 3.3.1 Inhibition of RAS: Single Agent of farnesyltransferase(FT) Inhibitor

One strategy to inhibit the function of RAS is through the inhibition of FT, as RAS must require lipid translation and modification to be localized to the membrane compartment. The most important posttranslational modification of RAS is that FT binds farnesyl to the cysteine residue at the carboxyl end of p21RAS. FT inhibitors prevent it from being carboxymethylated, thereby preventing the localization of p21RAS to the cell membrane and blocking the activation of RAS [107, 108, 109]. Tipifarnib (R115777) is an irreversible and selective FT inhibitor that has shown significant inhibition of cell growth in RAS mutation cell lines [110]. Lonafarnib is another tricyclic nonpeptidomimetic compound FT inhibitor [111]. In vitro, lonafarnib combined with MAPK pathway inhibitors had an additional cell growth inhibition of melanoma, and lonafarnib combined with sorafenib synergistically inhibited melanoma cell growth, pronouncedly enhanced apoptosis [112]. In the two Phase I studies, the recommended dose of tipifarnib is 300 mg bid for 21 days followed by 7 days off [113, 114]. Fourteen melanoma patients enrolled in a Phase II clinical trial (NCT00060125) of FT inhibitor had no treatment response and had been terminated prematurely [115].

Unsatisfactory clinical outcomes of FT inhibitors were developed for several mechanisms. First, FT inhibitors can inhibit the activation of T cells via T cell receptor (TCR) and interfere with T cell cytokine production [116]. Second, FT inhibitors can also affect other RAS family members (GTPases), such as Rheb, RAL, RhoC, and Rac1, which contribute to malignant transformation [117, 118, 119]. Third, FT inhibitors not only inhibit the RAS signing pathway, but also inhibit a plethora of molecules such as RET, ARHI, RERG, and RIG [108, 120]. Fourth, FT inhibitors are unable to block the binding of NRAS to cell membrane, as the geranylgeranyl transferase Type 1 (GGT1) can replace the function of FT [70, 115, 121]. However, a recent single‐arm Phase II trial (NCT02383927) enrolled head and neck squamous cell carcinoma with HRAS mutations (*n* = 22), and results showed tipifarnib had an encouraging efficacy with a median PFS of 5.6 months (95% CI, 3.6–16.4 months) and a median OS of 15.4 months (95% CI, 7.0–29.7 months) [122, 123]. The difference in response between subtypes of RAS may be attributed to the fact that KRAS and NRAS can escape FTI‐mediated inhibition by being prenylated by GGT1 and remaining fully functional, whereas HRAS is completely farnesylated [121, 124, 125].

#### 3.3.2 Inhibition of RAS: Combination Therapy of FT Inhibitor

S0438 (NCT00281957) was a randomized Phase II trial for untreated metastatic melanoma. In Arm B, 39 patients were given tipifarnib plus sorafenib; and the median PFS was 1.8 months and median OS was 7 months, with only one patient observed partial response (PR) [126]. In another Phase I study of tipifarnib in combination with erlotinib (EGFR TKI), two patients (two out of 27) had PRs including one advanced melanoma [127]. These trials suggest that combination therapy of tipifarnib also has limited antitumor effect in melanoma. In addition to FT inhibitor, considerable effort has gone into the targeting RAS in combination with GGT1 inhibitor. Single agent of GGT1 inhibitor has the ability to inhibit the RAS^mut^ tumor cells [128], and inhibition of both FT and GGT1 markedly reduced RAS‐driven tumor growth and completely inhibit the prenylation in the preclinical setting [125, 129, 130]. However, using doses of GGT1 inhibitors sufficient to block Ras prenylation was considered to be toxic [131]. To overcome GGT1‐related resistance to FT inhibitor, Kazi et al. designed FGTI‐2734, which is a dual FT and GGT1 inhibitor [132]. In vivo, FGTI‐2734 inhibited the growth of xenografts derived from four pancreatic cancer patients with KRAS (2 KRAS^G12D^ and 2 KRAS^G12V^) mutations. Further exploration of FT and GGT1 inhibitors, especially in combination therapy for NRAS mutant melanoma, is necessary for future research.

#### 3.3.3 Inhibition of RAS: Broad‐Spectrum RAS Inhibitor

Broad‐spectrum RAS inhibitors, such as RMC‐7977 and RMC‐6236, represent a new approach to target RAS mutations. RMC‐7977 is a reversible and highly selective tri‐complex (RMC‐7977–CYPA–RAS) RAS inhibitor, and it has affinity for both mutant and wild‐type RAS variants. In a preclinical study reported by Wasko et al., RMC‐7977 prominently inhibited a series of pancreatic ductal adenocarcinoma models with KRAS mutations via the RAS–MAPK pathway [133, 134]. For the near term, Holderfield et al. verified that RMC‐7977 not only inhibited KRAS^mut^, but also inhibited NRAS^mut^ (NRAS^Q61L^, NRAS^Q61K^, and NRAS^Q61R^) cancer cells [135]. RMC‐6236 has a similar structure to RMC‐7977 and exhibits potent inhibition of both KRAS^Q61^ and NRAS^Q61^ mutations [136]. A multicenter, dose escalation, Phase I/Ib trial of RMC‐6236 as a monotherapy for previously treated advanced solid tumors with KRAS^G12^ mutations, is ongoing (NCT05379985), as shown in Table 1 [136, 137]. These studies provide a strong preclinical rationale for the use of broad‐spectrum RAS inhibitors in RAS^mut^ tumors.

**TABLE 1:** Ongoing clinical trials of targeted therapy for NRAS mutation tumor.

Trial	Phase	Therapy	Targeted	Enrollment	Patient population	Primary outcome	Status
NCT03979651	Ib/II	Trametinib + hydroxychloroquine	MEK + autophagy	29	Advanced NRAS^mut^ melanoma	DLT, ORR	Unknown
NCT05340621	Ib/II	OKI‐179 + binimetinib	HDAC + MEK	36	RAS pathway (Ib); NRAS^mut^ melanoma (II)	DLT, AEs, ORR	Active
NCT03932253	Ia/Ib	FCN‐159	MEK	37	NRAS amplification and mutation (Ia) and NRAS^mut^ (Ib)	AEs, MTD, ORR	Unknown
NCT06008106	III	Tunlametinib vs. chemotherapy	MEK	2:1	NRAS^mut^ melanoma	PFS	Not yet recruiting
NCT02079740	Ib/II	Trametinib and navitoclax	MEK + BCL2	97	NRAS/KRAS^mut^ solid tumors	AEs, ORR, PFS	Active
NCT05585320	I/IIa	IMM‐1‐104	MEK	210	RAS^mut^ solid tumors	RP2D (I), AEs, DLT, ORR	Recruiting
NCT06229340	II	leflunomide + MEK inhibitor + hydroxychloroquine	MEK + autophagy	20	RAS^mut^ solid tumors	ORR	Recruiting
NCT06270082	I	IK‐595	MEK + RAF	150	RAS/RAF‐altered solid tumors	RP2D, AEs, DLT, MTD	Recruiting
NCT04439344	II	Binimetinib	MEK	53	NRAS solid tumors	ORR	Active
NCT02974725	Ib	Trametinib + LXH254	MEK + RAF	241	KRAS/BRAF^mut^ NSCLC or NRAS^mut^ melanoma	AEs, DLT	Active
NCT05907304	I	Trametinib + naporafenib	MEK + RAF	115	RAS^Q61X^ solid tumors	ORR	Recruiting
NCT03580382	Ib/II	Trametinib + CDX‐3379	MEK + ERBB3	3	NRAS^mut^ melanoma	ORR, OS	Terminated
NCT05580770	I/IIa	Mirdametinib + BGB‐3245	MEK + RAF	136	NRAS^mut^ melanoma	AEs, MTD, RP2D, ORR	Recruiting
NCT04109456	Ib	IN10018 + cobimetinib + atezolizumab	FAK + MEK + PD‐L1	120	UM or NRAS^mut^ melanoma	AEs	Recruiting
NCT04913285	I/Ib	KIN‐2787 + binimetinib	MEK + RAF	400	BRAF/NRAS^mut^ solid tumors	DLT, MTD	Recruiting
NCT04835805	Ib	Belvarafenib + cobimetinib + nivolumab	RAF + MEK + PD‐1	65	NRAS^mut^ melanoma	AEs, DLT	Active
NCT05379985	I/Ib	RMC‐6236	Pan‐RAS	472	RAS^mut^ solid tumors	AEs, DLT	Recruiting
NCT06026410	I	KO‐2806	FT	270	RAS^mut^ solid tumors	AEs, DLT, ORR	Recruiting
NCT04892017	I/II	DCC‐3116 ± trametinib/binimetinib	ULK + MEK	323	RAS^mut^ solid tumors	AEs, MTD, ORR	Recruiting
NCT05786924	I	BDTX‐4933	BRAF + RAS	100	Included NRAS^mut^ Melanoma	DLT, ORR, DOR, TTR, PFS	Recruiting
NCT03905148	Ib	Lifirafenib + mirdametinib	RAS + MEK	105	NRAS^mut^ solid tumors	AEs, ORR	Recruiting
NCT04249843	Ia/Ib	BGB‐3245	RAF	114	RAS^mut^ solid tumors	AEs, DLT, MTD, RP2D, ORR	Recruiting
NCT02407509	I	VS‐6766 ± everolimus	RAS + MEK	104	BRAF, KRAS, and/or NRAS^mut^ solid tumors	RP2D, AEs	Recruiting
NCT06096974	I	YL‐17231	Pan‐RAS	60	RAS^mut^ solid tumors	AEs, DLT	Not yet recruiting
NCT06208124	I/IIa	IMM‐6‐415	MEK	240	RAF/RAS^mut^ solid tumors	AEs, DLT, RP2D, ORR	Not yet recruiting
NCT05831995	I	ABM‐168	MEK	112	RAF/RAS/NF‐1^mut^ solid tumors	AEs, DLT, RP2D	Recruiting
NCT03454035	I	Ulixertinib + palbociclib	ERK + CDK4/6	45	RAS^mut^ solid tumors	MTD, ORR	Recruiting
NCT03875820	I	Defactinib + VS‐6766	RAF/MEK + FAK	87	RAS^mut^ solid tumors	AEs, DLT, RP2D	Active
NCT06270082	I	IK‐595	MEK/RAF	150	RAF/RAS^mut^ solid tumors	AEs, DLT, RP2D	Recruiting
NCT05554367	II	Palbociclib + binimetinib	CDK4/6 + MEK	199	RAS^mut^ solid tumors	PFS, ORR	Recruiting
NCT05111561	I	ZEN003694 + binimetinib	BET + MEK	42	RAS alteration solid tumors	DLT, AEs	Recruiting

*Data source*: Clinical data obtained from https://clinicaltrials.gov.

#### 3.3.4 Inhibition of RAS: RAS^G12C^ Inhibitor

RAS mutations have long been considered as untargetable, but sotorasib (AMG510) have shown success in KRAS^G12C^ mutation non‐small cell lung cancer [138, 139]. The glycine‐to‐cysteine mutation at position 12 (G12C) abnormally activates the KRAS protein, and the mutated cysteine locates next to the pocket of the Switch II region (P2). Sotorasib covalently binds to the P2 that present only in the inactive GDP binding form, trapping KRAS^G12C^ in an inactive state [140, 141]. In the NCT03600883 trial, an advanced melanoma patient harboring the KRAS^G12C^ mutation achieved a confirmed PR with a PFS of 5.6 months after treatment with sotorasib [142].

Unlike KRAS, the frequency of NRAS^G12C^ mutation is extremely low, with a mutation frequency of approximately 0.08% in all tumor types [143] and about 2% in all NRAS mutation [144]. In 2024, Rubinson et al. confirmed that sotorasib is a pan‐RAS^G12C^ inhibitors, and sotoraxib has a fivefold higher efficacy against NRAS^G12C^ compared with KRAS^G12C^ or HRAS [G12C^143^]. Other two KRAS^G12C^ inhibitors (JDQ443 and RMC‐6291) structurally related to sotorasib, also show strong inhibitory effects on NRAS^G12C^ mutation. Conversely, adagrasib, a KRAS^G12C^‐specific inhibitor, is relatively insensitive to NRAS^G12C^ mutation [143]. What is more, Rubinson et al. described a refractory and advanced colorectal cancer patient with NRAS^G12C^ mutation achieved a PR with sotorasib and panitumumab (anti‐EGFR antibody) in later‐line treatment [143]. In a case report, a metastatic (lung and liver) rectal cancer patient with NRAS^G12C^ mutation also responded to sotorasib and panitumumab, with a PFS of 4 months [145]. Thus, pan‐RAS^G12C^ inhibitors may offer a novel strategy for targeting NRAS^G12C^ mutations.

#### 3.3.5 Inhibition of RAS: MicroRNA

MicroRNA (miRNA) is a class of short regulatory RNA that participates in various aspects of tumor pathogenesis. In the context of melanoma, miR‐145‐5p has been identified as a tumor‐suppressive miRNA that directly targets NRAS. It can suppress the proliferation, invasion, and migration of melanoma cell lines by inhibiting MAPK and PI3K/AKT pathways, and induce apoptosis of melanoma cells [146, 147]. Another regulatory molecule, FUT8‐AS1 is a long noncoding RNA associated with the prognosis of melanoma [148]. It binds to nuclear factor 90 (NF90) directly and inhibits the interaction between NF90 and primary miR‐145‐5p. This promotes the production of miR‐145‐5p, upregulates mature miR‐145‐5p levels, and ultimately inhibits the NRAS–MAPK pathway [148]. The discovery of tumor‐suppressive miR‐708 has attracted the attention of researchers. In melanoma cell lines (SK‐MEL‐2) transfected with NRAS mutations, miR‐708 directly targets NRAS and inhibits cell proliferation and promotes apoptosis [149]. In 2005, Eskandarpour et al. used RNA interference technique to induce apoptosis in melanoma cells line with NRAS mutation [150]. Additionally, octenidine can form a G‐quadruplex structure in the untranslated region of NRAS mRNA, which downregulates NRAS translation, suppresses the MAPK and PI3K–AKT pathway, and leads to concomitant cell cycle arrest, apoptosis, and autophagy [151, 152].

#### 3.3.6 MAPK Pathway: MEK Inhibitors of Monotherapy

For advanced BRAF‐mutant melanoma, targeted therapies—such as the BRAF inhibitors dabrafenib and vemurafenib—have become standard treatment [100, 102, 153]. However, the signaling switch from BRAF to CRAF and RAF dimerization in RAS‐mutant melanoma has been identified as potential mechanisms of insensitivity for BRAF inhibitors [63, 154, 155].

Trametinib, the first US FDA‐approved MEK inhibitor for advanced melanoma, has achieved great success in combination with BRAF inhibitors for BRAF^mut^ melanoma. Previously, high expectations were placed on trametinib for NRAS^mut^ melanoma. According to the result from the Phase I trial (NCT00687622), trametinib obtained no objective response in 11 NRAS^mut^ patients, indicates that trametinib is not valid as a monotherapy for NRAS mutation melanoma [156]. The first MEK inhibitor recommend by National Comprehensive Cancer Network (NCCN) for the treatment of NRAS^mut^ melanoma was the small‐molecule MEK1/2 inhibitor binimetinib (MEK162). In an open‐label, nonrandomized, Phase II study (NCT01320085), binimetinib was tested for advanced melanoma harboring NRAS mutations. The results of MEK1/2 inhibitor seemed favorable with an ORR of 20% (six out of 30) and manageable adverse events [157]. The subsequent multicenter, randomized, Phase III trial (NEMO) compared the efficacy and safety of binimetinib to dacarbazine in patients with advanced NRAS‐mutant melanoma. Binimetinib significantly improved the median PFS (2.8 vs. 1.5 months, *p* < 0.001) but did not show a significant difference in median OS (11.0 vs. 10.1 months, *p* = 0.50) [158]. Phase II NCI‐MATCH study (NCT02465060) aimed to explore the efficacy of binimetinib in NRAS‐mutated cancers (without melanoma); among 47 patients in treatment population, the median PFS was 3.5 months, the median OS was 10.5 months, and the ORR was 2.3% [159].

**FIGURE 2:**
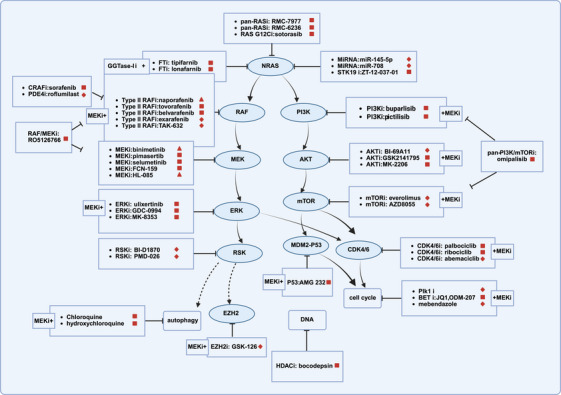
Targeted therapy for NRAS^mut^ melanoma. MEKi: MEK inhibitor; FTi: farnesyl transferase inhibitor; GGTase‐1 i: geranylgeranyl transferase Type 1 inhibitor. Red triangle: NMPA approved or NCCN recommend for NRAS^mut^ melanoma; red square: ongoing or completed clinical trials for NRAS^mut^ melanoma or other solid tumors; red diamond: preclinical stage; dashed line: indirect effect.

Up to now, several MEK inhibitors, including pimasertib, selumetinib, FCN‐159, and HL‐085, have been investigated for their efficacy and safety for NRAS‐mutant melanoma (Figure 2) [160, 161, 162, 163, 164, 165, 166]. Monotherapy with MEK inhibitors for NRAS^mut^ melanoma in the clinical trials is presented in Table 2. RO4987655 is another oral MEK inhibitor. Zimmer et al. enrolled 95 patients with advanced cancer with RAS/RAF mutations (including eight NRAS^mut^ melanoma) in the Phase I study (NCT00817518) of RO4987655. In the subgroup analysis, only one patient achieved PR and three patients achieved stable disease (SD) [167, 168]. More recently, Wei et al. enrolled 100 patients with advanced NRAS^mut^ melanoma and tested the efficacy and safety of the tunlametinib (HL‐085) in the Phase II study (NCT05217303). The results of tunlametinib were excellent, with a median PFS of 4.5 month, a median OS of 13.7 months, and a disease control rate (DCR) of 72.6% in 95 patients with central laboratory‐confirmed NRAS mutations (full analysis set) [169]. On March 15, 2024, tunlametinib became the first MEK inhibitor approved by the National Medical Products Administration (NMPA) of China for the treatment of advanced NRAS^mut^ melanoma.

**TABLE 2:** Clinical trials of MEK inhibitors for NRAS mutation melanoma.

Trial	Phase	Population	Patients	Therapy	Primary outcome	ORR	mPFS	Citations
NCT01320085	II	NRAS^mut^	30	Binimetinib	ORR	20% (6/30)	3.7 months (95% CI, 2.5–5.4)	[157]
NCT01763164	III	NRAS^mut^	269	Binimetinib	mPFS	15% (41/269)	2.8 months (95% CI, 2.8–3.6)	[158]
NCT05217303	II	NRAS^mut^	100	HL‐085	ORR	39.1%	4.2 months (95% CI, 3.5–5.6)	[169]
NCT03932253	Ia	NRAS^mut^	33	FCN‐159	AEs, MTD, ORR	19% (4/21)	4.8 months (95% CI, 2.8–not reached)	[160]
NCT03973151	I	NRAS^mut^	42	HL‐085	AEs, DLT, MTD	14.2% (6/42)	3.0 months (95% CI, 2.1–3.7)	[161]
NCT00982865	I	NRAS^mut^	93	Pimasertib	DLT	12.4% (11/89)	NR	[163]
NCT01693068	II	NRAS^mut^	130	Pimasertib	mPFS	23% (30/130)	13 weeks	[164]
NCT01143402	II	UM	50	Selumetinib	mPFS	14% (7/49)	15.9 weeks (95% CI, 8.4–21.1)	[165]
NCT00338130	II	BRAF/NRAS^mut^	45	Selumetinib	mPFS	11.1% (5/45)	NR	[166]

In general, single agent of MEK inhibitor, such as binimetinib, seems hard to achieve sustained responses with a mPFS of 2.8 months [158], and rapid resistance is the crucial problem, which leads to the failure of MEK inhibitors [170]. There are several approaches to improve the efficacy of MEK inhibitors: (1) combination therapy of MEK inhibitors (discussed further in more detail), (2) overcome primary and secondary resistance (discussed further in more detail), (3) intermittent therapy of MEK inhibitors. In 2020, Matter et al. first propounded intermittent MEK inhibitors treatment [171]. For one thing, melanoma cells switch phenotype into more invasive cell behavior under long‐term exposure to MEK inhibitor [172]. For another, short‐term MEK inhibition enriched CD8+ T cell in tumor microenvironment (TME) [173]

#### 3.3.7 MAPK Pathway: MEK Inhibitors Combined With RAF/ERK/RSK Inhibitors

Type I RAF inhibitors (GDC‐0879) and Type I½ ATP‐competitive RAF inhibitors (vemurafenib, dabrafenib, and encorafenib) are effective in BRAF^V600E/K^ melanoma, while these agents instead promote RAS‐dependent dimerization and activation of wild‐type RAF proteins in RAS^mut^ melanoma [174, 175]. Thus, it is not surprising that the combination of trametinib and low‐dose Type I RAF inhibitor (dabrafenib) failed in Phase II trial (TraMel‐WT, NCT04059224) for advanced NRAS^Q61R/K/L^ melanoma [176]. Previous studies demonstrated that CRAF‐mediated signaling plays a significant role in reducing the sensibility of MEK inhibitors, and targeting CRAF in combination with MEK inhibitors has been explored as a strategy to improve the efficacy [177]. Although ablating CRAF alone is not sufficient to block tumor growth, proliferation depends on ARAF‐mediated ERK activation [178].

Type II RAF inhibitors are considered to be pan‐RAF inhibitors, which targeted the unique “DGF‐out” and “αC‐helix‐in” conformations of the kinase, and at least five inhibitors (naporafenib, tovorafenib, belvarafenib, exarafenib, and TAK‐632) showed activity in BRAF^mut^ or RAS^mut^ melanoma [179, 180, 181]. Naporafenib (LXH254) is a novel Type II pan‐RAF kinase inhibitor that prevents BRAF and CRAF dimerization [182, 183, 184]. In the expansion arm (*n* = 30) of Phase Ib study (NCT02974725), naporafenib was explored in combination with trametinib in patients with NRAS‐mutant melanoma. The mPFS was 5.03 months in 15 patients that underwent naporafenib 200 mg twice per day plus trametinib 1 mg once daily, and 4.21 months in another 15 patients treated with naporafenib 200 mg once a day plus trametinib 1 mg once daily [183]. Belvarafenib (Type II inhibitor) showed antitumor effects in patients with NRAS‐mutant melanoma in the Phase I trials (NCT02405065, NCT03118817). In the dose‐escalation cohort of belvarafenib, nine NRAS‐mutant melanoma patients achieved four PR, with a median PFS of 25 weeks. In the dose‐expansion cohort, 10 patients with NRAS‐mutant melanoma achieved two PR and four SD [179, 185]. A recent Korean study has revealed the value of belvarafenib in BRAF or NRAS mutation melanoma patients [186]. In contrast, tovorafenib, another pan‑RAF inhibitor, did not show responses in the Phase I NCT01425008 study in patients with NRAS^mut^ melanoma (*n* = 17) and nine patients had a best response of SD [187].

Atefi et al. observed that pan‐RAF plus MEK inhibitors had a significant growth inhibition in the majority of the NRAS‐mutant melanoma cell lines; meanwhile, these inhibitors exhibited high levels of synergism [188]. However, in addition to inhibit RAF, off‐target kinase inhibition of these pan‐RAF inhibitors can limit dose escalation. Their toxicities are suggestive of receptor tyrosine kinase inhibition including rash and blood creatine phosphokinase increased [183]. In order to reduce toxicity, researchers have developed a new Type II ATP‐competitive of highly selective RAF dimer inhibitor RAF709 [189]. RAF709 presents a regression of patient‐derived xenografts of NRAS^mut^ melanoma with well tolerance [190]. RO5126766 (also known as VS‐6766 and CH5126766) is a highly selective dual RAF and MEK inhibitor [191, 192, 193]. It induces G1 cell cycle arrest by upregulation of the CDK inhibitor p27 and downregulation of CCND1 [194]. In the Phase I dose‐escalation study (NCT00773526), the dose recommended for Phase II (RP2D) investigation was 2.7 mg, and observed one PR in two patients with NRAS mutation [192]. RO5126766 was tested in the dose escalation and basket dose‐expansion Phase I study (NCT02407509) in patients with RAS/RAF‐mutant solid tumors. The study demonstrated that RO5126766 has antitumor activity and is tolerant to RAS/RAF‐mutant tumors, with 27% (seven out of 26) patients achieved objective responses [193]. Overall, the dual inhibition of MEK and pan‐RAF may be more effective than single‐agent therapy with MEK inhibitor or pan‑RAF inhibitor.

MEK inhibitors can activate phospho‐ERK (pERK) in the NRAS‐mutant melanoma cell lines, and activation of pERK leads to cyclin‐D1 expression and therapeutic escape [170]. ERK inhibitors can inhibit the emergence of resistance and overcome acquired resistance to MEK inhibitors as demonstrated by several studies [195, 196, 197, 198, 199, 200, 201]. Ulixertinib (also named as BVD‐523), an ERK1/2 kinase inhibitor, the RP2D was 600 mg twice a day [195, 202, 203], has been investigated the pharmacokinetics, safety, and preliminary efficacy in advanced solid tumor patients with MAPK mutation in a Phase I trial (NCT01781429) [195]. The initial data showed that ulixertinib with 600 mg twice a day have a promising antitumor effect (17 NRAS^mut^ melanoma in the dose‐expansion cohorts including three had PR, six had SD) and manageable toxicities. In 2024, ulixertinib did not show activity in patients with metastatic uveal melanoma in a Phase II study [204]. GDC‐0994, another inhibitor of ERK1/2, demonstrated its safety in Phase I dose‐escalation study (NCT01875705) but showed limited efficacy (ORR: 4%, two out of 47) of patients with advanced solid tumors [205]. The combination of MEK and ERK inhibitors suppresses the expression of cyclin‐D1, enhances the apoptosis, and inhibits the growth of BRAF mutant, NRAS mutant, and wild‐type melanoma [170, 197, 206, 207]. In 2013, Morris et al. have identified that SCH772984 (ERK1/2 inhibitor) may help overcome the resistance of BRAF‐mutant melanoma to BRAF or MEK inhibitor [197]. However, the combination of ERK inhibitor (MK‐8353) and MEK inhibitor (selumetinib) did not demonstrate the antitumor activity for metastatic solid tumors at tolerable doses in the Phase Ib study (NCT03745989) [208]. Similarly, the combination of cobimetinib (MEK inhibitor) plus GDC‐0994(ERK1/2 Inhibitor) showed limited antitumor activity (only one patient had an unconfirmed PR) and was associated with Grade 3 dose‐limiting toxicities (DLTs) in a Phase Ib study (NCT02457793) in patients with advanced solid tumor (*n* = 24) [209]. As of now, there have been no completed clinical trials reporting the safety and efficacy for the combination of MEK and ERK inhibitors for NRAS^mut^ melanoma.

RSK is the central downstream effector of MAPK signaling and is activated by ERK1/2 [67]. RSK promotes G2 DNA damage checkpoint silencing by phosphorylating checkpoint kinase 1 (Chk1) at Ser280, and inhibition of RSK can sensitize melanoma cells to DNA‐damaging chemotherapy [210]. Previous RSK inhibitors, such as BI‐D1870, have shown antitumor effects in vitro. However, these inhibitors are unstable and have a poor pharmacokinetic profile, which hinder their application in vivo [211, 212]. The novel pan‐RSK inhibitor PMD‐026 has been overcame these limitations and improved oral bioavailability [213]. In a preclinical study, Kosnopfel et al. reported single agent of RSK inhibitor (PMD‐026) significantly impaired NRAS^mut^ melanoma cell in vitro and suppressed the growth of tumor in vivo [214]. Currently, only one ongoing Phase I/Ib clinical trial (NCT04115306) test the PMD‐026 for metastatic breast cancer.

#### 3.3.8 MAPK Pathway: MEK Inhibitors Combined With PI3K/mTOR/AKT Inhibitors

Crosstalk occurs between MAPK pathway and PI3K pathway is one of the reasons that lead to MEK inhibitor resistance [215, 216], and PI3K pathway is another critical signaling pathway in NRAS‐mutant melanoma [65, 66]. The PI3K family consists of three different Classes I–III, classified based on primary structure, regulation, and lipid substrate specificity in vitro. Class I PI3Ks have a catalytic subunit with four isoforms, including PIK3CA (alpha), PIK3CB (beta), PIK3CG (gamma), and PIK3CD (delta) [217, 218]. Buparlisib is a potent and selective oral pan‐Class I PI3K inhibitor [218, 219]. Aasen et al. observed that combined therapy with buparlisib and trametinib was more effective than monotherapy, more effectively increased apoptosis in vitro, and inhibited tumor growth in vivo [220]. In an open‐label, single‐arm, Phase II trial (NCT02452294), Amaral et al. investigated the safety and efficacy of monotherapy with buparlisib in patients with asymptomatic brain metastasis of melanoma who had received at least one systemic therapy [219]. In this study, single agent of buparlisib was well tolerated but limited antitumor activity, with no intracranial responses. Pictilisib (GDC‐0941) is another potent and highly specific inhibitor of the Class I PI3K (pan‐PI3K inhibitor) and showed its safety in the dose‐escalation study (NCT00876122) [221].

Although monotherapy of pan‐PI3K inhibitor did not show a clinical benefit in the I/II study, several studies demonstrated that inhibition of MEK and PI3K/mTOR had antitumor activity and synergistic effects in vitro and in vivo [66, 222, 223, 224]. The results of Phase Ib study (NCT01155453) showed that PI3K inhibitor (buparlisib) in combination with the trametinib for patients with advanced solid tumors (including six NRAS^mut^ solid tumors) had a well antitumor activity at RP2D, while treatment‐related adverse events (TRAEs) lead to frequent dose interruptions [225]. In another Phase Ib study (NCT01363232), binimetinib in combination with buparlisib was investigated in the patients with advanced solid tumors with RAS/RAF alterations. However, 32 out of 89 patients discontinued treatment due to the TRAEs, suggesting that toxicity maybe a limiting factor for the combination of MEK and PI3K inhibitors [226]. The efficacy and tolerability of pan‐PI3K inhibitor (pictilisib) plus MEK inhibitor (cobimetinib) were also disappointing in patients with solid tumors in the NCT00996892 study [227].

Small molecule PI3K inhibitors typically also inhibit mTOR (mTORC1 and mTORC2; discussed further in more detail) due to the structurally similar p110 subunits of PI3K and mTOR [228]. Pan‐PI3K/mTOR inhibitor, such as omipalisib and voxtalisib, may have therapeutic advantages by providing more efficient inhibition of the PI3K/AKT/mTOR pathway. BEZ‐235 (pan‐PI3K–mTOR inhibitor) or omipalisib combined with as‐700026 (MEK inhibitor) has shown the most pronounced synergistic effect in inducing apoptosis and inhibiting proliferation of melanoma cell lines [229]. However, the combination therapy of omipalisib and trametinib in the Phase Ib dose‐escalation study (NCT01248858) was poorly tolerated, and minimal responses were observed in melanoma patients (including two SD and three PD) [230]. Similar results were observed in a Phase Ib study of pan‐PI3K/mTOR inhibitor combined with MEK inhibitor (NCT01390818: voxtalisib + pimasertib) [231]. The significant toxicity of the combination therapy (pan‐PI3K inhibitor + MEK inhibitor or pan‐PI3K/mTOR inhibitor + MEK inhibitor) is likely due to several factors. The main reason is that both of inhibitors did not selectively for mutant kinases, and long‐term inhibition of MAPK and PI3K–AKT–mTOR pathway influence normal cellular biological processes, leading to tolerability concerns [232]. So, reducing the overlapping toxicities of pan‐PI3K/mTOR and MEK inhibitors will be crucial for future clinical research in NRAS^mut^ melanoma.

AKT is a serine/threonine protein kinase with three types (isoforms) AKT1, AKT2, and AKT3 that participate in multiple signing pathways including survival, proliferation, and distant metastasis of melanoma [233, 234]. Small molecule inhibitors, such as BI‐69A11, can inhibit all three isoforms of AKT and suppress the cell viability of melanoma with NRAS mutation in vitro and the growth of xenografts tumor models [235, 236]. GSK2141795 is another potent, selective, ATP‐competitive and oral pan‐AKT inhibitor that decreases phosphorylation of various AKT substrates and inhibits cell proliferation in vitro [237, 238]. In the dose‐escalation Phase I study of NCT00920257, GSK2141795 was found to be well tolerated, with a RP2D of 75 mg daily for patients with solid tumors [237]. Other small‐molecule AKT inhibitors, such as MK‐2206, have also demonstrated safety in the several Phase I studies [239, 240, 241, 242].

The combination of MEK and AKT inhibitors can be highly sensitive in NRAS^Q61K^ melanoma cell lines in a preclinical study [243]. In clinical, combining MEK inhibitors with AKT inhibitors has been associated with a high level of TRAEs, although most of them are tolerable when the dose was reduced [244, 245, 246]. The efficacy of MEK inhibitors combined with AKT inhibitors is also not ideal [247]. In the Phase II study (NCT01941927), the combination of trametinib and GSK2141795 does not have significant clinical activity in NRAS‐mutant melanoma, no objective responses were observed, and the mPFS and mOS were only 2.3 months (95% CI, 2.1–2.5 months) and 4.0 months (95% CI, 0.9–7.0 months) [248]. These results may reveal different issues: (i) the R2PD of the combination therapy are lower than the dose of monotherapy, (ii) the combination therapy was not well tolerated, and (iii) the heterogeneity and disease stage of NRAS^mut^ melanoma may lead to the negative outcomes.

mTOR is a serine/threonine protein kinase regulating many important intracellular processes and has two functionally separate protein complexes (mTORC1 and mTORC2) [249, 250]. Both everolimus (mTORC1 inhibitor) and AZD8055 (mTORC1 and mTORC2 inhibitor) sufficiently block the cell growth of NRAS^mut^ neuroblastoma cell lines [222]. In the same way, inhibition of mTOR (rapamycin and everolimus) had a significant impact on cell cycle, cell proliferation, and invasive potential of human melanoma cell lines [251, 252]. In the NRAS^mut^ melanoma cell lines, although MEK inhibitor plus PI3K inhibitor had a superior inhibition than the combination of MEK and mTOR inhibitors (mTOR inhibition can activate upstream AKT [253]), MEK inhibitor plus mTOR inhibitor also have an effective inhibition of the cell growth and regression of xenograft model [65]. The combination therapy of MEK inhibitor and mTOR inhibitor had an acceptable tolerability in several prospective Phase I clinical trials such as NCT00955773 and NCT01378377 [254, 255].

Phosphoinositide‐dependent kinase‐1 (PDPK1) is a key kinase downstream of PI3K that activates AGC kinases and oncogenic signaling pathways such as the AKT pathways [256, 257, 258]. Suppressing PDPK1 combined with MEK inhibitor (trametinib) treatment decreased cell proliferation in MEK inhibitor‐resistant cells, increased the CD8+ T cells in the TME, and suppressed tumor growth [259].

In the future, targeting both the CAF/MEK/ERK and PI3K/AKT/mTOR might serve as a therapeutic option for the treatment of NRAS‐mutant melanoma. However, the biggest challenge for the combination therapy is the DLT, which prevents the establishment of optimal therapeutic concentrations [246].

#### 3.3.9 MAPK Pathway: MEK/RAF Inhibitors Combined With Cell Cycle Inhibitors/Apoptotic Regulators

Both MAPK and PI3K pathway can upregulate CCND1, and cell cycle regulation is a hallmark of malignant tumors including NRAS‐mutant melanoma [68, 260]. In clinical, several CDK4/6 inhibitors, including PD0332991 (palbociclib), LEE011 (ribociclib), and LY2835219 (abemaciclib), were approved by US FDA for antitumor therapy [261, 262, 263, 264, 265, 266]. In the Phase II study of NCT01320085, NRAS‐mutated patients with CCND1 or CCND3 alterations had a shorter mPFS in the treatment of binimetinib, implying that the CDK4/6 pathway signaling may lead to resistance of MEK inhibitors [157, 267].

The preclinical studies have shown the antitumor activity of cotargeting MEK and CDK4/6, which created excitement for oncologists. In vitro and in vivo, CDK4/6 inhibitor (palbociclib) showed combination effects with MEK inhibitor in RAS‐driven NSCLC cell lines, including increased growth inhibition and the population of cells in G1 phase, and overcome the resistant to the MEK inhibitor [268, 269]. Similar results have been observed in CDK4/6 inhibitor (abemaciclib) combined with MEK1/2 inhibitor (trametinib) in A375 melanoma cell line (NRAS wild‐type) [270]. Posch et al. observed antitumor effect of MEK and CDK4/6 inhibitors in vitro and in melanoma xenograft models [271]. Schedules of continuous MEK with intermittent CDK4/6 inhibitors led to more tumor responses than other combination schedules [272]. MEK inhibitor alone induced robust apoptosis but failed to engage the cell cycle checkpoint; CDK4/6 inhibitor treatment alone suppressed proliferation but failed to impact on apoptosis, and the combination of MEK and CDK4/6 inhibitors resulted in both apoptosis and cell cycle arrest in the xenograft of NRAS^Q61K^ mutation melanoma [273]. Hayes et al. suggested that NRAS‐mutant melanomas can upregulate the activity of both RAF and PI3K pathways and lead to the acquired resistance of MEK1/2 and CDK4/6 inhibitors [274].

Single agent of abemaciclib partially inhibited phosphorylation of RB1 and increased CCND1, and LY3009120 (RAF inhibitor) combined with abemaciclib has the ability to completely inhibit RB1 phosphorylation and suppress abemaciclib‐mediated upregulation of CCND1. RAF inhibitor and CDK4/6 inhibitor showed a synergistically inhibition for NRAS mutation melanoma in vitro and in vivo [275]. In the Phase I/II multicenter study (NCT02202200), Louveau et al. established the maximum tolerated dose (MTD) of palbociclib and vemurafenib, showed a mPFS of 2.8 months, an estimated ORR of 27.8% (five out of 18), and an estimated DCR of 83.3% (15 out of 18) in BRAF^mut^ metastatic melanoma [276]. Another Phase I–II study (NCT01781572) tested ribociclib plus binimetinib in patients with NRAS‐mutant melanoma. The results were positive, the combination therapy is safe, and the RP2D is binimetinib 45 mg twice daily and ribociclib 200 mg once daily; the ORR of Phase II cohort (*n* = 41) was 19.5%, and the mPFS and mOS of all patients were 3.7 months (95% CI, 3.5–5.6 months) and 11.3 months (95% CI, 9.3–14.2 months), respectively [277]. This study suggested that combined MEK inhibitor with CDK4/6 inhibitor may be clinically more active in NRAS^mut^ melanoma patients with concurrent genetic alterations (such as CDKN2A, CDK4, or CCND1) in G1 cell‐cycle checkpoint than MEK inhibition alone. Different from MEK plus RAF/ERK/PI3K inhibitors, the combination of MEK inhibitor and CDK4/6 inhibitor seemed to be safe and well tolerated, and TRAEs were consistent with single agents supporting a lack of drug interaction between the two inhibitors.

P53 is a classic tumor suppressor gene, while Webster et al. observed that inhibited p53 can restore the sensitivity of melanoma cells to MEK inhibitors [278]. And it was reported that p53 driven by Wnt5A induce a slow‐cycling state of melanoma cell, which led to the resistance of MEK inhibitors. AMG 232, a potent and selective inhibitor of the MDM2–p53 protein, activates TP53 signaling, which resulted in significantly superior antitumor efficacy and regression, and markedly increased activation of p53 signaling in vitro [279]. AMG 232 showed a mild/moderate AEs in solid tumors and multiple myeloma in the study of NCT01723020, and an acceptable safety in acute myeloid leukemia when combined with or without trametinib in the study of NCT02016729 [280, 281]. In the Phase I study (NCT02110355), AMG 232 was investigated for safety and efficacy in combination with MAPK inhibitors (dabrafenib plus trametinib or trametinib alone) in metastatic wild‑type TP53 melanoma. Ten patients with BRAF^mut^ melanoma and 21 patients with BRAF wild‐type melanoma were enrolled in the Arm 1 and Arm 2. The mPFS of Arm 1 and Arm 2 was 19.0 and 2.8 months, respectively. Three melanoma patients with NRAS mutation observed tumor regression and the best efficacy was SD [282].

Previous studies have shown the therapeutic potential of targeting antiapoptotic pathways in melanoma [283, 284, 285, 286, 287]. Polo‐like kinase 1 (Plk1) is a mitotic regulator, which is related to cell cycle regulation, apoptosis and malignant transformation, and overexpression in primary NRAS‐mutant melanoma and melanoma metastases [287, 288, 289]. Inhibition of MAPK pathway leads to a decrease in Plk‐1 expression, which is a consequence of G1 cell‐cycle arrest rather than direct regulation of Plk‐1 [289]. MEK plus Plk1 inhibitors resulted in an additive growth reduction of NRAS‐mutant melanoma in vitro and vivo. The potential mechanisms underlying the efficacy of MEK/Plk1 inhibitors combination therapy include enhanced CHK/p53 pathway, arrest of cells in both the G0/G1(induce by MEK inhibitor) and G2/M (induce by Plk1 inhibitor) phase, and eventually induction of apoptosis in NRAS‐mutant melanoma cell [287, 290].

#### 3.3.10 MAPK Pathway: MEK Inhibitors Combined With Epigenetic Modulators

The bromodomain and extra terminal domain (BET) family, including BRD2, BRD3, BRD4, and BRDT, is a key epigenetic regulatory factor, which is overexpressed in melanoma cells, regulating DNA replication and cell cycle progression [291, 292, 293, 294]. Vargas et al. evaluated the efficacy of BET inhibitor combined with MEK inhibitor for NRAS^mut^ melanoma. The combination therapy of BRD4 inhibitor (JQ1) plus MEK inhibitor (PD901) synergistically downregulated the transcription factor 19 (TCF19), increased apoptosis in vivo, reduced the growth of NRAS‐mutant melanoma, and prolonged the survival of mice which resisted to MAPK inhibitors and ICIs [295]. NCT03035591, an open‐label and dose‐escalation Phase I study, evaluated the safety, pharmacokinetics, MTD, and preliminary antitumor activity of the ODM‐207 (pan‐BET inhibitor) in solid tumors. The result showed that ODM‐207 was safe in dose escalation [296]. Another pan‐BET inhibitor, mivebresib (ABBV‐075), has been tested in the Phase I dose‐escalation study (NCT02391480) in patients with advanced solid tumors including 10 patients (14%) with uveal melanoma. No PR was observed in all of the patients, SD was observed in 25 patients, and tumor shrinkage was observed in only 11 patients including four uveal melanomas [297]. Furthermore, Phase I study (NCT05111561) is recruiting patients with solid tumors that carrying RAS alterations to test the BET inhibitor (ZEN003694) in combination with binimetinib.

Histone deacetylases (HDACs) belong to the epigenetic regulatory enzymes family, which regulate chromatin structure by removing acetyl groups on histone lysine tails, resulting in a more compact chromatin structure and enhancing transcriptional activity [298, 299]. Singel agent of HDACs inhibitor (panobinostat) is not active in the treatment of metastatic melanoma in a Phase I study [300]. Maertens et al. explored the combining of HDAC inhibitors (entinostat) with BRAF/MEK inhibitors in melanoma cells harboring BRAF, NRAS, PTEN, or NF1 mutations. Specifically, in NRAS^mut^ and NF1^mut^ melanoma cell lines, the combination of entinostat with MEK inhibitors has shown to enhance tumor regression via triggering the cooperatively suppression of nonhomologous end‐joining genes, leading to a chemical synthesis lethality caused by excessive DNA damage [301]. For uveal melanoma, the combination of HDAC inhibitors and MEK inhibitors has greater efficacy than monotherapy, as HDAC inhibition suppressing GPCR‐mediated YAP activation and RTK‐driven AKT signaling, which are considered key pathways for rapid resistance for MEK inhibitors [302]. The coadministration of HDAC inhibitors and MEK inhibitors significantly suppressed the cell viability of KRAS^mut^ pancreatic cancer cells [303]. Bocodepsin (OKI‐179) is a potent inhibitor of Class I HDACs (1, 2, 3, and 8) [304] and showed significant antitumor activity in several human cancer cell lines in preclinical studies [304, 305]. The safety, MTD, RP2D, and efficacy of bocodepsin is evaluated in a Phase I trial (NCT03931681) [306]. Bocodepsin combined with binimetinib is currently being evaluated in patients with NRAS^mut^ melanoma in the Phase Ib/II Nautilus trial (NCT05340621). The preliminary results from this trial showed an ORR of 37.5% (six out of 16), a DCR of 75% (12 out of 16), and no Grade 4/5 TRAEs reported.

Histone H3 lysine 27 trimethylation, which was downregulated by Enhancer of Zeste Homolog 2 (EZH2), has been identified as a key role in the invasive/mesenchymal regulators of NRAS mutation melanoma [307, 308]. Also, activated RAS signaling leads to EZH2 overexpression through MAPK–ERK1/2–EIK1 and PI3K–AKT pathways [309, 310]. EZH2 inhibition has ability to induce a shift of H3K27 from trimethylation to acetylation, and EZH2 inhibitor (GSK‐126) showed to markedly reduce invasiveness and significantly suppress the tumor growth in NRAS^mut^ melanoma when combined with trametinib [307, 311]. EZH2 inhibitor plus MEK inhibitor provide a promising therapeutic strategy for NRAS^mut^ melanoma.

#### 3.3.11 MEK Inhibitor Combined With Autophagy Inhibitor

Chloroquine, a classical antimalarial drug that inhibits lysosomal acidification, is commonly used as an inhibitor of autophagy in vitro [312, 313]. Chloroquine can promote apoptosis and reduce the growth of melanoma xenografts by degrading the antiapoptotic PUMA protein in BCL‐2 family [312, 314]. Kinsey et al. hypothesized that trametinib‐induced autophagy maybe a mechanism for the secondary resistance to RAF–MEK–ERK inhibitors, and subsequent in vivo experiments confirmed this hypothesis by showing the synergistic antiproliferative effects of MEK1/2 inhibitors combined with chloroquine on patient‐derived xenografts of NRAS‐mutated melanoma [315]. Another antimalarials drug, mefloquine, has also shown efficacy in inducing cell death in NRAS mutation melanoma cell by endoplasmic reticulum and redox stress responses [316]. Based on the preclinical study that chloroquine can prevent autophagy‐driven resistance and improve the efficacy of BRAF and MEK inhibitors, Awada et al. tested the hydroxychloroquine, dabrafenib, and trametinib for BRAF‐mutant melanoma patients previously treated with BRAF/MEK inhibitors and ICIs in the Phase II study. The ORR was 20.0% (two out of 10) and the DCR was 50.0% (five out of 10) [317]. The combination of hydroxychloroquine, dabrafenib, and trametinib in the treatment of BRAF‐mutant melanoma was tested in another prospective Phase I/II trial (BAMM, NCT02257424). The 1‐year PFS rate was 48.2%, ORR was 85.0%, mPFS was 11.2 months (95% CI, 5.4–16.9 months) and mOS was 26.5 months (95% CI, 9.3–43.7 months). Three‐drug combination therapy had well tolerance, with a low rate of Grade 3 toxicities and no Grade 4 toxicities observed [318]. Based on the results of these two trials, the safety of hydroxychroroquine combined with MEK inhibitors has been validated and holds promise for improving outcome of NRAS‐mutant melanoma.

#### 3.3.12 Alternative Ways to Enhance MEK Inhibitor Effects

Focal adhesion kinase (FAK) promotes the aggressive phenotype of melanoma cells such as invasion and migration [319, 320, 321]. The researches of late indicated that FAK activate both MAPK pathway and PI3K–mTOR pathway [322, 323], while its role has not been confirmed in NRAS^mut^ melanoma. NCT01938443 study was a Phase Ib study that tested the combination of GSK2256098 (FAK inhibitor) and trametinib for patients with mesothelioma or other solid tumors with probable MAPK pathway activation [324]. The combination therapy showed a controllable safety profile, with a median PFS of 11.8 weeks. What is more, FAK inhibitor IN10018 was evaluated as monotherapy and then in combination with cobimetinib in a Phase Ib clinical trial (NCT04109456) for NARS mutation melanoma. The preliminary results showed that MEK inhibitor plus FAK inhibitor has an ORR of 32.5% and a median PFS of 5.45 months [325].

Phosphodiesterase Type 4 (PDE4) is a cAMP‐specific enzymes that essential for the conversion from BRAF to CRAF in NRAS mutation melanoma through inhibiting cAMP signaling [64]. PDE4D (one subtype of PDE4) has been found to interact with FAK and promote BRAF^mut^ melanoma invasion through the scaffolding protein RACK1 [326]. The preclinical study demonstrated that the combination of roflumilast (PDE4 inhibitor) and cobimetinib (MEK inhibitor) can suppress the proliferation of patient‐derived NRAS^Q61^ melanoma in vitro [327]. Further investigations are needed to clarify the PDE4 inhibitor for NRAS^mut^ melanoma in the future.

#### 3.3.13 MEK Inhibitor Combined With Repurposed Drugs, Chemotherapy, and Radiotherapy

Several cardiac glycosides, including digoxin and digitoxin, have been found to exhibit increased toxicity to melanoma cells compared with normal human melanocytes in vivo [328]. Based on these observations, a Phase IB trial of NCT02138292 investigated the digoxin and trametinib in advanced BRAF wild‐type melanoma including six patients with NRAS^mut^ melanoma [329]. Surprisingly, two of six NRAS mutation patients had PR (ORR: 33.3%).

Mebendazole is a classic anthelmintic agent with disruptive microtubule effects, as well as anticancer effect, such as inhibiting mitotic spindles, inducing G2/M arrest and apoptosis [330]. The combination of mebendazole and MEK inhibitor (trametinib) dramatically inhibited ERK1/2 phosphorylation, suppressed the ERK1/2‐mediated pathways, and decreased expression of BCL2 in NRAS^Q61K^ melanoma cell lines [331]. In xenografts of NRAS^Q61K^ melanoma, the combination therapy also strongly inhibited the growth of tumor.

Chemotherapy is a traditional strategy for the treatment of melanoma, and the efficacy of chemotherapy for NRAS^mut^ melanoma is not well established. In the PACMEL trial, a Phase I dose‐escalation trial of trametinib in combination with weekly paclitaxel, enrolled 15 melanoma patients without BRAF mutations. The mPFS and mOS were 5.5 months (95% CI, 1.8–7.8 months) and 14.1 months (95% CI, 4.6 months–not reached), respectively. Four patients harboring NRAS mutations had PRs, with an ORR of 50% (four out of eight) [332]. Double‐blind multicenter Phase II trial (DOC‐MEK, NCT01256359) randomized (1:1) patients with wild‐type BRAF melanoma to docetaxel with selumetinib or placebo [333]. This study enrolled 83 patients, and 37 patients harboring NRAS mutation, 23 patients with NRAS wild‐type, and 17 patients unknown of mutation. For whole population, the median PFS was 4.23 months in the selumetinib group and 3.93 months in the placebo group (*p* = 0.130). Subgroup analysis showed that NRAS mutation status did not significantly impact the median PFS. Therefore, there were no evidence to support the combination of chemotherapy and MEK inhibitor for NRAS mutation melanoma [333].

In the treatment of melanoma, radiotherapy is primarily used as adjuvant therapy for patients at risk of lymph node field recurrence [334, 335] or as palliative therapy for patients with symptomatic disease such as brain and bone metastasis [336]. On the other way, ionizing radiation has been confirmed to activate the MAPK and PI3K/AKT pathway [337, 338]; meanwhile, NRAS mutations lead to intrinsic resistance of tumor cells to radiotherapy through induction of EGF and the DNA damage response [339]. In the real‐world, NRAS mutation was an independent predictor for radiotherapy local control of melanoma brain metastases [82]. Consequently, MEK inhibitor combined with radiotherapy has a potential synergistic effect. After the combination therapy of radiotherapy and selumetinib, tumor cells (A549, MiaPaCa2, and DU145) present as impaired activation of the G2/M checkpoint and mitotic catastrophe [340]. Shannon et al. reported that selumetinib and radiotherapy (10 Gy/5f) inhibit the tumor growth of xenografts (Calu‐6 cell) by reducing the level of hypoxia‐inducible factor‐1 alpha, GLUT‐1, and vascular endothelial growth factor [341]. The synergistic effect of trametinib and radiotherapy was also observed in NRAS/BRAF mutation melanoma cell line (D04, WM1631) [342]. The NCT00970359 study enrolled 20 patients with advanced thyroid cancer who received the combination therapy of selumetinib and iodine‐131 (20 Gy) [343]. In the NRAS^mut^ population (*n* = 5), four patients had confirmed PRs and one patient had confirmed SD. Additional clinical trials investigating the combination treatment of MEK inhibitors and radiotherapy are warranted.

#### 3.3.14 Mutitarget tyrosine kinase inhibitors(TKIs)

NRAS^Q61^‐mutated melanoma relies on glucose metabolism, and glucose deprivation promotes the conversion of CRAF to BRAF, leading to BRAF‐mediated phosphorylation of PFKFB2/PFKFB3, key glycolytic enzymes that mediate the response to metabolic stress. PFKFB2 is associated with PFK1 activation, leading to sustained production of fructose 1,6‐bisphosphate and ultimately feedback to RAS signaling [63, 344, 345]. The combination of 2‐deoxy‐d‐glucose (glycolysis inhibitors) and sorafenib (multikinase TKI, which inhibits CRAF, BRAF, VEGFR‐2, VEGFR‐3, PDGFR‐β, Flt3, c‐Kit, and p38α^67^) significantly suppresses the tumor growth of patient‐derived xenografts of NRAS^Q61^ melanoma [344]. In a preclinical study, sorafenib has demonstrated the inhibition of the MAPK pathway in KRAS^mut^ or BRAF^mut^ colon and pancreatic cell lines [67]. In vivo, sorafenib inhibited tumor growth of BRAF^V600E^ mutation melanoma xenograft models [346]. Flaherty et al. designed a a Phase I dose‐escalation trial, armed to test the safety, MTD, and antitumor activity of sorafenib in combination with carboplatin and paclitaxel [347]. In this study, 24 melanoma patients were enrolled, including nine patients with BRAF mutation and two patients with NRAS mutation. One PR and one SD occurred in two NRAS^mut^ melanoma patients, and one CR, four PR, and three SD were observed in nine BRAF^mut^ melanoma patients [347]. However, the subsequent Phase II trials showed limited antitumor activity and manageable side effect of sorafenib monotherapy in patients with advanced melanoma [348, 349]. In the study of NCT00119249, only one patient achieved PR in 36 patients with sorafenib monotherapy [349]. The randomized, Phase III, ECOG 2603 study (NCT00110019) enrolled 823 patients and analyzed 179 patients (41 patients harboring NRAS mutation), and compared the treatment of carboplatin plus paclitaxel with or without sorafenib [350]. For patients with NRAS mutant melanoma, the median PFS was 3.0 and 5.1 months for nonsorafenib and sorafenib arms (*p* = 0.079), respectively, and the median OS was 9.8 and 10.3 months for nonsorafenib arms (*n* = 18) and sorafenib arms (*n* = 22; *p* = 0.506), respectively, the ORRs were 5.6 and 22.7% for nonsorafenib and sorafenib arms [350]. Mechanistically, sorafenib improved the ORR and median PFS may be due to the inhibition of CRAF, which plays a crucial role in activating the MAPK pathway in NRAS^mut^ melanoma [63].

Fedorenko and his colleague presented a preclinical study for NRAS‐mutant melanoma with amuvatinib, which is a multikinase inhibitor targeting mutant Kit, c‐MET, PDGFR‐α, and Rad51 [351, 352]. They analyzed the enriched gene of NRAS‐mutant melanoma cell lines, and the results showed that the overexpression of Axl, c‐KIT, and c‐MET, which as the target for amuvatinib. In the vitro, amuvatinib inhibited the growth of NRAS‐mutant melanoma cell by inducing G2/M‐phase cell cycle arrest and apoptosis, but not BRAF‐mutant melanoma cell lines [351].

Lenvatinib is a novel multitarget TKI which inhibits RET, VEGFR1–3, FGFR1–4, KIT, and PDGFRα [353, 354]. Unlike sorafenib, lenvatinib lacks targets for the CRAF or MAPK signaling pathways. NCT00121680 was a Phase Ib clinical trial and evaluated the DLT, safety, pharmacokinetics, and efficacy of lenvatinib in combination with temozolomide for metastatic or unresectable melanoma patients [355]. In the highest combination dose (lenvatinib 24 mg + temozolomide 150 mg/m^2^), the clinical benefit of NRAS mutation melanoma (*n* = 5) included two PR and three SD [355]. Another dose‐escalation Phase I study assessed the DLT, MTD, safety, and response of lenvatinib monotherapy for patients with advanced melanoma in the expanded cohort [356]. NRAS mutations were detected in eight patients, and the results were encouraging, with an ORR of 12.5% (one out of eight) and a DCR of 87.5% (seven out of eight) [355].

### 3.4 Beyond MAPK: Targeting Alternative Pathways

#### 3.4.1 NF1

The NF1 protein functions as a negative regulator of RAS proteins via its GTPase activity [357]. Germline mutations in NF1 cause neurofibromatosis Type I, a RASopathy characterized by dysregulation of the RAS/MAPK pathway. In melanoma, NF1 mutations occur in approximately 12–18% of all cases but are markedly enriched in the desmoplastic melanoma (45–93%). NF1‐mutant melanomas are clustered within elderly individuals and chronically sun‐exposed skin, and they exhibit a distinctive molecular profile marked by high mutation burden and the absence of BRAF or NRAS mutations [358, 359]. Given that NF1 mutations primarily enhance RAS/MAPK signaling, therapeutic inhibition of this pathway is a rationally supported strategy for NF1‐mutant melanoma. Inhibitors of the PI3K/mTOR pathway and cell cycle are also promising for NF1‐mutant melanoma, as discussed in Section 3.3.

#### 3.4.2 MITF Pathway

MITF is the production of antiapoptotic Bcl‐2 gene, and it also related to the survival of melanocytes and melanoma cells [360]. As a key regulator of melanocyte differentiation and pigmentation, MITF is essential for melanocyte homeostasis. Notably, approximately 20% of melanomas exhibit MITF amplification. BRAF exerts dual control over MITF in melanoma. While BRAF‐activated ERK signaling targets MITF for ubiquitin‐dependent degradation, it concurrently stimulates transcription factors that increase MITF expression. The presence of both BRAF mutations and MITF amplification in 10–15% of cases indicates that the degradation of MITF by ERK is more complex and is likely influenced by additional regulatory factors [29, 361]. Meanwhile, the dysregulation of MITF appears of critical importance for melanoma drug resistance [362, 363]. The strong activation of MITF/Bcl‐2 pathway is one of reasons that lead to acquired resistance of MEK inhibitor for NRAS‐mutant melanoma, and anti‐Bcl‐2 (ABT‐199) annihilated the acquired resistance and restored the sensitivity of NRAS‐mutant melanoma cells to MEK inhibitor [364].

#### 3.4.3 WNT Pathway

The pursuit of therapeutic targets is further complicated by the intricate interplay between WNT signaling and other pathways essential for melanoma pathogenesis, notably MAPK/ERK and PI3K/AKT pathways [365, 366]. The mechanism involving combined BRAF inhibition and WNT/β‐catenin activation involved AXIN1 degradation and GSK3β inhibition, while hyperactivation of the MAPK/ERK pathway stabilized AXIN1 and consequently inhibited WNT signaling in melanoma [365]. Mutations in key WNT pathway genes APC, AXIN1, and CTNNB1 occur at low frequencies in melanoma (10, 2.9, and 5.9%, respectively, as reported by cBioPortal) [367]. In preclinical development, various WNT pathway inhibitors have been explored. Key signaling components, particularly Frizzled receptors (FRZDs) and Dishevelled, have been validated as targets for small‐molecule inhibition. The therapeutic antibody vantictumab (OMP‐18R5) acts by interfering with WNT–FRZD binding and has shown tumor growth inhibition in multiple human xenograft models, with enhanced efficacy in combination with standard chemotherapy [29].

#### 3.4.4 NTRK

NTRK fusions, which result from rearrangements in the NTRK1, NTRK2, or NTRK3 genes (encoding TRKA, TRKB, and TRKC, respectively), are actionable oncogenic drivers found in a diverse range of adult and pediatric cancers. NTRK fusions are uncommon in melanoma overall. However, they are notably identified in Spitz melanomas, accounting for a substantial proportion (approximately 21%) of these tumors [368]. Furthermore, NTRK translocations have also been identified in 2.5% of acral melanomas and less than 1% of cutaneous and mucosal melanomas [369, 370]. Targeting NTRK fusions is a promising strategy, supported by selective NTRK inhibitors such as entrectinib and larotrectinib, which are US FDA approved for adult and pediatric patients with metastatic or unresectable, NTRK‐rearranged solid tumors who have no satisfactory alternative treatments. The NTRK inhibitors entrectinib and larotrectinib have demonstrated a 57 and 79% ORR, respectively, in tumors with NTRK fusions, regardless of histology [371, 372]. Given this efficacy, these predictive biomarkers would be worth testing in tumors from patients diagnosed with metastatic melanomas.

## 4 Overcoming Therapeutic Resistance: Mechanisms and Strategies

### 4.1 Resistance Mechanisms of BRAF Inhibitor

Only part of BRAF mutation melanoma patients can benefit from BRAF inhibitor, and these patients with objective response may rapidly develop secondary resistance. The most common mutations are BRAF splice variants, BRAF amplification, secondary BRAF mutations, NRAS mutations, and MEK1/2 mutations. Also, the rate of resistance development does not appear to be related to the antitumor activity of the BRAF inhibitor.

Splice variants of BRAF lack exons 4–8 containing RBD, which mediates resistance by affecting BRAF dimerization. In cells with wild‐type BRAF, activation by RAS leads to the formation of homodimers (BRAF–BRAF) or heterodimers with CRAF (BRAF–CRAF), whereas cells with V600E mutations do not form dimers and activate MEK via monomeric BRAF. Splice variants of BRAF V600E are also able to form dimers in a RAS‐independent manner and therefore to activate MEK in the presence of BRAF inhibitors. The amplification of the BRAF gene led to significant upregulation of BRAF protein expression, contributing to the reactivation of ERK in the presence of BRAF inhibitors [373]. Secondary mutations in V600E (single‐nucleotide substitution encoding L505H) have been detected in patients with BRAF inhibitor resistance [374, 375]. The mutations in V600E increases BRAF kinase activity and causes cross‐resistance with MEK inhibitors. Secondary NRAS mutations and MEK1/2 mutations activate MAPK pathway and are thus predicted to diminish response to MAPK inhibitors [95, 373, 376].

### 4.2 Resistance Mechanisms of MEK Inhibitor

#### 4.2.1 Intrinsic Resistance

For intrinsic resistance of MEK inhibitors, Appleton et al. demonstrated that abnormal activation of Rho/MRTF pathway is associated with high intrinsic resistance to trametinib in the melanoma cell lines with NRAS mutation. Combining trametinib with the Rho/MRTF‐pathway inhibitor, CCG‐222740, synergistically reduced the viability of trametinib‐resistant cells in vitro [199]. ROCK1 and ROCK2 are highly homologous Rho‐associated protein kinase that regulate migration, invasion, and metastasis [200]. Single agent of ROCK inhibitor has shown the suppression of the tumor growth of wild‐type melanoma xenograft models [201], and the combination of ROCK and MEK inhibitors has been exhibited to promote apoptosis of NRAS^mut^ melanoma in vivo and inhibit tumor proliferation in vitro [202].

#### 4.2.2 Acquired Resistance

Acquired resistance to MEK inhibitors involves signals from NRAS, CRAF, COT, PDGFRβ, EGFR, and IGF‐1R, as well as reactivation of ERK1/2 [154, 377, 378]. Meanwhile, reactive oxygen species (ROS) can reactive ERK1/2, which is located at the center of MAPK pathway, indicating that ROS plays a crucial role in the resistance of BRAF and MEK inhibitors in BRAF mutation melanoma [379, 380]. However, ROS seems to play a completely opposite role in melanoma with NRAS mutations. Eichhoff et al. reported that the combination of ROS inducer and MEK inhibitor suppresses both tumor growth and metastasis of NRAS^mut^ cell lines in vivo and overcomes resistance of MEK inhibitors [381]. ERK5, another member of the MAPK family, highly homologous to ERK1/2, contains a kinase domain and a transcriptional transactivation domain. MEK5–ERK5 pathway is involved in the several pathogeneses including cell survivals, antiapoptosis, proliferation, and angiogenesis of melanoma [382, 383]. Several studies have demonstrated that the resistance of MEK inhibitors (trametinib, binimetinib, selumetinib, and cobimetinib) and ERK1/2 inhibitors lead to the abnormal activation of ERK5 [384, 385, 386]. Inhibition of MEK and ERK5 (BIX02189) can suppress the activity of resistance melanoma cells in vitro and the growth of tumor in vivo [386, 387].

Activation of the metabolic enzyme phosphoglycerate dehydrogenase (PHGDH) has been reported as the factors lead to the secondary resistance to MEK inhibitors [364]. PHGDH is a crucial metabolic enzyme in the serine biosynthesis pathway [388, 389, 390], and upregulation of PHGDH resulted in increase of tricarboxylic acid cycle, increase of glutathione production, and the promotion of tumor growth [388, 391, 392]. Suppression of PHGDH can resensitize MEK inhibitor‐resistant NRAS^mut^ melanoma cells to trametinib treatment, increase the ability of cellular to combat oxidative stress, and decrease glutathione production and cell proliferation [393].

## 5 Integration of Targeted Therapy and Immunotherapy

Given the lack of satisfying target therapies for NRAS mutation melanoma and the proven efficacy of immunotherapy for melanoma, immunotherapy is considered as another potential strategy for NRAS/BRAF mutation melanoma. We focus on the clinical data of ICIs and optimized combination methods of ICIs and MEK inhibitors in NRAS/BRAF mutation melanoma.

### 5.1 Overview of Immunotherapy for Melanoma

#### 5.1.1 ICIs

In the last decade, ICIs have changed the therapeutic model of advanced melanoma [394, 395, 396, 397, 398]. A randomized Phase III trial (KEYNOTE‐006) compared the efficacy of pembrolizumab (a PD‐1 inhibitor) given every 3 weeks, pembrolizumab given every 2 weeks, and ipilimumab (a CTLA‐4 inhibitor). Median PFS of three groups were 5.5 months (95% CI, 3.4–6.9 month), 4.1 months (95% CI, 2.9–6.9 month), and 2.8 months (95% CI, 2.8–2.9 month), respectively [399, 400, 401]. In the Phase III trial CheckMate‐067, nivolumab (a PD‐1 inhibitor) monotherapy or in combination with ipilimumab significantly improved the mPFS, mOS, and ORR compared with ipilimumab in patients with metastatic melanoma [402, 403, 404]. To this date, pembrolizumab, ipilimumab, and nivolumab have been approved by US FDA for advanced melanoma. In China, pembrolizumab, toripalimab, and pucotenlimab have been approved by NMPA for advanced melanoma.

It is still controversial whether NRAS‐mutant metastatic melanoma is more responsive to ICIs (Table 3). Guida et al. enrolled 162 NRAS‐mutant/BRAF wild‐type and 169 NRAS/BRAF wild‐type patients, which underwent ICIs. Regarding the outcomes to ICIs, no significant differences were reported in ORR (42 vs. 37%), DCR (60 vs. 59%), mPFS (12 vs. 9 months), or mOS (32 vs. 27 months) of these two cohorts [405]. In another retrospective study, anti‐CTLA‐4 plus anti‐PD‐1 had similar ORR (40 vs. 39%, *p* = 0.578), DCR (61 vs. 73%, *p* = 0.125), mPFS (4 vs. 3 months), and mOS (32 vs. 37 months, *p* = 0.630) between NRAS‐mutant melanoma and NRAS wild‐type melanoma [406]. Joanna et al. reported BRAF/NRAS mutation status did not influence the outcome of patients treated with ipilimumab alone due to no significant difference of the mOS (10.12 vs. 8.26 months, *p* = 0.67) [407]. Recently, in a multicenter study, Zaremba et al. implied that NRAS mutational status did not impact mPFS, mOS, and ORR in patients treated with ICIs [408]. In 2023, Panning et al. reported that NRAS, BRAF^V600E^, NF1, and triple‐negative mutations were not associated with significant difference of median PFS and median OS in 73 advanced melanoma patients treated with ICIs [409].

**TABLE 3:** Studies of ICIs for NRAS mutation melanoma.

Source	Patients	Therapy	Line	mPFS	mOS	ORR	DCR
Guida et al. [405]	162 NRAS^mut^, 169 NRAS^wt^	ICIs	Any	12 vs. 9 months, *p* = 0.51	32 vs. 27 months, *p* = ns	42 vs. 37%, *p* = 0.38	60 vs. 59%, *p* = 0.90
Kirchberger et al. [406]	125 NRAS^mut^, 53 NRAS^wt^	Ipilimumab	Any	NM	12 vs. 27 months, *p* = 0.046	15 vs. 13%, *p* = 0.731	27 vs. 40%, *p* = 0.101
	34 NRAS^mut^, 8 NRAS^wt^	Anti‐PD‐1	Any	3 vs. 4 months	18 vs. 30 months, *p* = 0.077	21 vs. 13%, *p* = 0.210	35 vs. 25%, *p* = 0.578
	77 NRAS^mut^, 67 NRAS^wt^	Ipilimumab + anti‐PD‐1	Any	NM	32 vs. 37 months, *p* = 0.630	40 vs. 39%, *p* = 0.859	61 vs. 73%, *p* = 0.125
Mangana et al. [407]	24 NRAS^mut^, 38 BRAF^mut^, 39 BRAF/NRAS^wt^	Ipilimumab	Any	NM	12.1 vs. 8.03 vs. 8.26 months, *p* = 0.56	NM	NM
Zaremba et al. [408]	184 NRAS^mut^, 197 NRAS^wt^	Anti‐PD1	First	9.0 vs. 12.4 months, *p* = 0.21	27.9 vs. 29.4 months, *p* = 0.97	26.1 vs. 35%	42.4 vs. 53.8%
	99 NRAS^mut^, 93NRAS^wt^	Anti‐PD1 + CTLA‐4	First	29.4 vs. 24.2 months, *p* = 0.48	42.3 months vs. NR, *p* = 0.14	32.4 vs. 34.4%	47.6 vs. 53.8%
	27 NRAS^mut^, 37 NRAS^wt^	Anti‐CTLA‐4	First	3.7 vs. 2.6 months	37.6 vs. 37.6 months	18.5 vs. 10.8%	29.6 vs. 21.6%
Johnson et al. [410]	60 NRAS^mut^, 169 NRAS^wt^	ICIs	Any	4.1 vs. 2.9 months, *p* = 0.08	19.5 vs. 15.2 months, *p* = 0.51	32 vs. 20%, *p* = 0.07	50 vs. 30%, *p* < 0.01
Rose et al. [411]	69 NRAS^mut^	Anti‐PD‐1 + anti‐CTLA4 vs. anti‐PD1	Any	Not reached vs. 7.0 months	Not reached vs. 21.9 months	NM	NM
Dupuis et al. [412]	15 NRAS^mut^, 51NRAS^wt^	Anti‐PD‐1	Any	15.1 vs. 3.9 months, *p* = 0.2	66 vs. 53.4% (1 year OS rate), *p* = 0.32	NM	NM
van Not et al. [413]	498NRAS^mut^, 699 BRAF^mut^, 567 BRAF/NRAS^wt^	Anti‐PD‐1	Any	8.1 vs. 9.8 vs. 11.7 months	23.6 vs. 42.5 vs. 28.5 months, *p* < 0.001	53 vs. 55 vs.56% (first line)	NM
	215NRAS^mut^, 303 BRAF^mut^, 241 BRAF/NRAS^wt^	Ipilimumab + nivolumab	Any	4.8 vs. 9.9 months, (*p* = 0.016) vs. 5.3 months	14.2 vs. not reached vs. 16.1, *p* < 0.001	48 vs. 59 vs. 45% (first line)	NM
Zhou et al. [418]	21 NRAS^mut^, 71NRAS^wt^ (cutaneous melanoma)	Anti‐PD‐1	Any	2.7 vs. 7.0 months, *p* = 0.024	13.8 vs. 20.4 months, *p* = 0.081	9.5 vs. 23.9%, *p* = 0.223	47.6 vs. 66.2%, *p* = 0.123
	12 NRAS^mut^, 102NRAS^wt^ (noncutaneous melanoma)	Anti‐PD‐1	Any	3.6 vs. 4.3 months, *p* = 0.015	10.8 vs. 15.3 months, *p* = 0.025	0 vs. 13.7%, *p* = 0.356	33.3 vs. 51.0%, *p* = 0.247
Panning et al. [409]	13NRAS^mut^, 22 BRAF^mut^	ICIs	Any	20.3 vs. 24.0 months	25.5 vs. 25.4 months	NM	NM
Shoushtari et al. [417]	40NRAS^Q61^, 48 BRAF^V600E^, 51NF1^mut^, 42TN	Anti‐PD‐1	Any	4.2 vs. 7.5 vs. 22 months vs. not reach, *p* < 0.001	NM	NM	NM

Abbreviations: TN, triple negative; NM, not mention.

Another viewpoint believed that NRAS^mut^ melanoma had superior outcomes compared with the BRAF^mut^ melanoma and NRAS/BRAF wild‐type of immunotherapy. Johnson et al. enrolled 60 patients with NARS^mut^ melanoma, 53 patients with BRAF^mut^ melanoma and 116 patients with NRAS/BRAF^wt^ melanoma. The NRAS‐mutant patients had superior or marginally statistical significance in ORR (28 vs. 16%, *p* = 0.04), DCR (50 vs. 31%, *p* < 0.01), and mPFS (4.1 vs. 2.9 months, *p* = 0.09) compared with the non‐NRAS mutation patients in any line of immunotherapy [410]. Similar results were observed in another retrospective study reported by Rose et al. NRAS mutation significantly improved mPFS (HR = 0.34, 95% CI, 0.16–0.71, *p* = 0.004) and mOS (HR = 0.24, 95% CI, 0.10–0.62, *p* = 0.003) when treated with PD‐1 plus CTLA4 inhibitors in the multivariable analyses [411]. A retrospective study reported by Dupuis et al. and the results showed NRAS‐mutated had trends toward better ORR and PFS than NRAS wild‐type without statistical significance [412].

While others studies had conflicting results, they believed that patients harboring NRAS mutation had a poor outcome [413, 414, 415, 416, 417]. In a national retrospective study of Dutch, 1764 enrolled patients received PD‐1 inhibitor and 759 received ipilimumab plus nivolumab in the first‐line treatment. In the group of ipilimumab plus nivolumab, mPFS was significantly higher for melanoma with BRAF mutation (9.9 months; 95% CI, 6.8–17.2 months) compared with NRAS‐mutant (4.8 months; 95% CI, 3.0–7.5 months) and BRAF/NRAS wild‐type (5.3 months; 95% CI, 3.6–7.1 months) [413]. In Asian population of a systemic meta‐analysis, NRAS mutation melanoma had shorter mPFS (3.6 vs. 4.3 months, *p* = 0.015) and mOS (10.8 vs. 15.3 months, *p* = 0.025) than NRAS wild‐type melanoma [418]. Cutaneous melanoma and noncutaneous melanoma with NRAS mutation have different outcome in Asian population; the mPFS, mOS, and DCR were 2.7 (95% CI, 1.7–3.7 months), 13.8 months (95% CI, 3.7–23.9 months), and 47.6% in the cutaneous melanoma (*n* = 21); and the mPFS, mOS, and ORR were 3.6 months (95% CI, 0.9–6.3 months), 10.8 months (95% CI, 1.5–20.1 months), and 33.3% in the noncutaneous melanoma (*n* = 12), respectively [418]. In a study of adjuvant PD‐1 inhibitor therapy for Stage III melanoma, there were 23 patients (20%) that harbored BRAF mutation, 21 patients (18.3%) harbored NRAS mutation, and 59 patients (51.3%) with BRAF/NRAS wild‐type [414]. NRAS^mut^ melanoma patients showed the worst outcome with a median disease‐free survival (DFS) of 9 months (*p* < 0.0001 vs. wild‐type) than BRAF^mut^ (median DFS: 17 months, *p* = 0.022 vs. wild‐type) and BRAF/NRAS wild‐type (median DFS: 32 months) [414]. NRAS mutation was considered as a poor outcome factor for monotherapy of PD‐1 inhibitor (toripalimab) from the results of the Phase II POLARIS‐01 trial (NCT03013101) with a mPFS of 1.8 months and an ORR of 6.3% [419, 420]. In our previous study of adjuvant PD‐1 inhibitors for acral melanoma, we enrolled eight patients with NRAS mutation and 23 patients without NRAS mutation. The median relapse‐free survival of NRAS^mut^ melanoma was shorter than NRAS wild‐type melanoma (5.9 vs. 18.9 months, *p* = 0.08) [415].

Why do PD‐1 inhibitors have completely conflicting outcomes for NRAS^mut^ melanoma in several studies? The main reason maybe the different types of melanomas. First, European and American researchers mainly enrolled patients with cutaneous melanoma, while Asian researchers mainly included enrolled acral and mucosal melanoma [405, 408, 415, 418]. Second, we observed a decrease in the abundance of myeloid dendritic cells (DCs) in the TME of NRAS mutation acral melanoma, while this phenomenon was not observed in cutaneous melanoma [415]. Third, patients previously received MEK inhibitors also influence the efficacy of PD‐1 inhibitors [406, 421]. Finally, most studies were small sample, single institution, and retrospective studies, which inevitably lead to bias. Up to now, no prospective clinical trials evaluated single agent of PD‐1 inhibitor for this unique subtype of melanoma.

Lymphocyte‐activation gene 3 (LAG‐3) is another immune checkpoint expression on immune cells (including T cells, NK cells, and Tregs) and it negatively regulates T‐cell proliferation and effector T‐cell function when LAG‐3 binds to major histocompatibility complex class II (MHC II) ligands [422, 423, 424, 425]. LAG‐3 and PD‐1 are commonly coexpressed on CD8+ TILs, thus contributing to tumor‐mediated immune suppression [424, 426, 427], and blocking both receptors can synergistically reduce tumor growth and strengthen antitumor immunity [426]. In the prospective clinical trial (RELATIVITY‐020), anti‐LAG‐3 combined with anti‐PD‐1 was considered as a safety strategy for advanced melanoma [428]. In the Phase II/III, double‐blind, randomized RELATIVITY‐047 study (NCT03470922), the researchers evaluated relatlimab (LAG‐3 blocking antibody) combined with nivolumab compared with nivolumab alone to patients with previously untreated advanced melanoma [429]. The mPFS was 10.1 months (95% CI, 6.4–15.7 months) with relatlimab + nivolumab and 4.6 months (95% CI, 3.4–5.6 months) with nivolumab; *p* = 0.006. In the subgroup analysis, a benefit of mPFS was also observed in dual inhibition of LAG‐3 and PD‐1 over PD‐1 alone in BRAF^mut^ melanoma, and the NRAS mutation status has not been revealed. Based on the “game changing” results, relatlimab combined with nivolumab therapy was approved in 2022 by the US FDA for the treatment of advanced melanoma [430].

#### 5.1.2 ICIs Combined With Antiangiogenic Therapy

VEGFR inhibitors have a synergistic effect with ICIs by enhancing tumor infiltration of immune cells and reducing the immuno‐suppressive effects of Tregs and myeloid‐derived suppressor cells (MDSCs) [431, 432, 433, 434, 435]. Also, VEGFR inhibitor plus ICIs have demonstrated the efficacy and safety in various solid tumors [436, 437, 438, 439, 440]. Axitinib, a highly selective VEGFR inhibitor, is approved for the treatment of advanced renal‐cell carcinoma [441, 442]. In a Phase Ib trial, axitinib in combination with toripalimab (a humanized monoclonal PD‐1 antibody) was tested for metastatic mucosal melanoma [443]. The combination treatment was tolerable and showed favorable antitumor activity in patients with metastatic mucosal melanoma. In this study, six patients with NRAS^mut^ underwent the combination therapy, including five patients achieved PR and one patient achieved SD [443]. In a cohort of 14 patients with NRAS^mut^ advanced mucosal melanoma from a real‐world study, axitinib plus PD‐1 inhibitor (toripalimab or pembrolizumab) demonstrated clinically meaningful antitumor activity, with an ORR of 62.5% (five out of eight) in the first‐line therapy [444]. Further, the combination of lenvatinib and pembrolizumab showed an ORR of 48% (10 out of 21), a mPFS of 5.5 months, and a median duration of response (DOR) of 12.5 months in the metastatic melanoma cohort (*n* = 21) in the Phase Ib/II NCT02501096 trial [445]. In the LEAP‐004, a single‐arm Phase II study (NCT03776136), 103 melanoma patients who previous progression on anti PD‐1/PD‐L1 were enrolled and treated with lenvatinib plus pembrolizumab. The mPFS and mOS were 4.2 months (95% CI, 3.8–7.1 months) and 14.0 months (95% CI, 10.8—not reached), respectively. However, in the population of BRAF mutation melanoma, irrespective of prior treatment with BRAF/MEK inhibitors, the ORR of combination therapy was only about 10% [446]. Apatinib is a small molecule, multitarget TKIs which could potently inhibit VEGFR‐2, c‐kit, and c‐src [447]. The nonrandomized, single‐arm, single‐center Phase II CAP 03 study of apatinib in combination with camrelizumab (PD‐1 inhibitor) and temozolomide (NCT04397770) enrolled a total of 50 patients with advanced acral melanoma in the first‐line treatment [448]. Findings of the study showed that the ORR was 64.0%, the median PFS was 18.5 months, and the median OS was not reached in total population, and it was worth mentioning the ORR of NRAS^mut^ patients (*n* = 14) was 64.3%.

#### 5.1.3 ICIs Combined With Chemotherapy

Previous studies have demonstrated chemotherapy can promote the antitumor immunity through several mechanisms [449, 450, 451, 452, 453, 454]. Paclitaxel can directly inhibit MDSCs, which have the ability to potently suppress T cell responses [454]. So, there is a good rationale to combine ICIs with chemotherapy. The Phase II trial (NCT01676649) examined the safety and efficacy of ipilimumab plus carboplatin/paclitaxel for advanced melanoma (NRAS^mut^: *n* = 2, BRAF^mut^: *n* = 8, NRAS/BRAF^mut^: *n* = 1, NRAS/BRAF^wt^: *n* = 19) [455].Mutations in NRAS/BRAF were more frequently observed in patients with PD compared with the patients who achieved clinical benefit (nine out of 17 vs. two out of 13, *p* = 0.034).

Nab‐paclitaxel is an albumin‐bound form of paclitaxel that enhance the antitumor effects while reducing the toxicity of paclitaxel [456]. Previous study demonstrated that RAS mutated cell metabolizes of extracellular albumin as energy supplements by enhancing macropinocytosis [457, 458, 459]. Therefore, Nab‐paclitaxel may take advantage of the unique mechanism of macropinocytosis for NRAS‐mutant melanoma therapy. In the real‐world, the combination of Nab‐paclitaxel and ICIs showed well efficacy. In a Chinese retrospective study, nab‐paclitaxel combined with anti‐PD‐1 inhibitor improve the outcomes of NRAS^mut^ melanoma patients in the first‐line therapy [8]. Similarly, Zhang et al. reported the combination therapy significantly improved the DCR of 100% in NRAS^mut^ melanoma compared with 54.5% in NRAS^wt^ melanoma (*p* = 0.03) [458].

#### 5.1.4 ICIs Combined With HDAC Inhibitors

HDAC inhibitors showed a synergistic effect in combination with the immune‐activating antibodies target CD40 and CD137 in multiple tumors cell lines [460, 461]. HDAC inhibitors mediated tumor cell apoptosis, decreased the expression of FOXP3 and HELIOS in Tregs, upregulated PD‐L1 expression and Class I/II human leukocyte antigen (HLA), decreased Tregs and MDSCs, and increased CD8+ TILs in tumor, which indicate that the combination of HDAC inhibitors and immunotherapy maybe an efficacy strategy for solid tumor [462, 463, 464]. In 2022, a Phase 1b study (NCT03565406) explored the safety, tolerability, and clinical efficacy of mocetinostat (HDAC Inhibitor) combined with nivolumab plus ipilimumab [465]. In general, the combination therapy had favorable response rates in 10 patients with advanced melanoma, while simultaneously accompanied by high levels of toxicity including six patients had Grade 3–4 toxicities [465].

#### 5.1.5 High‐Dose IL‐2

High‐dose interleukin‐2 (IL‐2) has been approved as an immunotherapy regimen for metastatic melanoma since 1998. It was initially approved due to the durable disease remissions observed in a small part of patients, although the regimen is associated with relatively high toxicity and treatment‐related deaths (1–2%) [466]. Different mutation status in patients with metastatic melanoma treated with high‐dose IL‐2 had a statistically significant difference in the response rate (CR or PR) in a multicenter retrospective study. NRAS^mut^ melanoma patients had a response rate more than twice that of BRAF‐mutant or NRAS/BRAF wild‐type patients (47 vs. 19%, *p* = 0.04). Additionally, patients with NRAS mutations had longer mPFS (214 vs. 70 days, *p* = 0.13) and mOS (5.3 vs. 2.4 years, *p* = 0.30), although the differences did not reach statistical significance [467]. Despite the availability of numerous approved regimens for metastatic melanoma, high‐dose IL‐2 remains an important treatment option. In a retrospective analysis, high‐dose IL‐2 therapy showed durable antitumor activity in metastatic melanoma patients (*n* = 40) who previously received PD‐1 or PD‐L1 inhibitors. The ORR was 22.5% (nine out of 40), with four CRs and five PRs [468]. High‐dose IL‐2 represents a treatment option in patients with metastatic melanoma after first‐line ICIs‐based immunotherapy and BRAF/MEK‐targeted therapy [469]. High‐dose IL‐2 sequenced with vemurafenib was investigated for BRAF^mut^ metastatic melanoma in a multicenter Phase II study (NCT01683188). Patients of Cohort 1 (*n* = 38) received vemurafenib for 6 weeks before high‐dose IL‐2 and patients of Cohort 2 (*n* = 15) received vemurafenib for 7–18 weeks before enrollment, both of Cohort 1/2 received high‐dose IL‐2. However, the ORR (27 vs. 27%), DCR (77 vs. 73%), 1‐year survival (60 vs. 53%), 2‐year survival (46.7 vs.40%), and 3‐year survival (30 vs. 26.7%) were similar between two cohorts; and Cohort 2 had a longer mPFS than Cohort 1 (8.3 vs. 5.3 months, *p* = 0.087) [470]. Systemic HD IL‐2 may upregulate Treg function and lead to immunosuppression, which could explain why the combination treatment regimen did not achieve the expected synergistic effect, and another Phase II study demonstrated it [471].

#### 5.1.6 Cancer Vaccines

Cancer vaccines are another immunotherapeutic approach which induce an effective immune response against tumor antigens.

A Phase I study used granulocyte–macrophage colony‐stimulating factor as an adjuvant and intradermal immunization with an NRAS peptide (residues 49–73) containing four codon 61 mutations [472]. In this study, two of 10 patients detected response to the vaccine in vitro, suggesting vaccine potential efficacy in triggering a T cell response against the mutant NRAS peptides [472]. Anti‐EGFR monoclonal antibodies (cetuximab and panitumumab) combined with BRAF inhibitors have shown safety and efficacy in metastatic colorectal harboring BRAF mutations [473, 474]. Additionally, a preclinical study has shown that anti‐EGF vaccination antibodies enhanced the activity of MAPK/ERK and PI3K/AKT inhibitors and reduced the proliferative effects of NRAS mutation melanoma cell lines [475]. These findings support the clinical exploration of combining MAPK/ERK inhibitors with anti‐EGF vaccination in BRAF/NRAS^mut^ melanoma.

Neoantigens are different from the somatic mutation of wild‐type antigens, as it arises from unique mutations specific to each individual patient and stimulates a highly specific immune antitumor activity, thus personalized neoantigen cancer vaccines are a fully personalized immunotherapy [476, 477]. A peptide‐based neoantigen vaccine called EVX‐01 has tested the safety, immunity, and preliminary efficacy for metastatic melanoma in the NTC03715985 study [478]. This Phase I study enrolled untreated metastatic melanoma patients (Group A), and melanoma patients who received PD‐1 inhibitor for more than 4 months with SD (Group B). EVX‐01 demonstrated the ability to trigger persistent specific T cell responses in all melanoma patients, and durable tumor responses were observed, even at the lowest dose level of the vaccine. The following and ongoing Phase II trial of KEYNOTE—D36 arms to evaluate the safety and efficacy of EVX‐01 in combination with pembrolizumab for advanced melanoma. These Phase I/II studies potentially pave the way for future Phase III trials in metastatic melanoma (including NRAS mutation melanoma) [479].

#### 5.1.7 Adoptive Cell Therapy

Adoptive cell therapy (ACT), including chimeric antigen receptor T cells (CAR‐T) therapy, TCR T cells therapy, and TILs therapy, is a kind of immunotherapy that involves collecting T cells from patients, modifying them in vitro, and then transfusing in vivo to enhance the ability to kill cancer cells [480, 481]. TILs recognize specific antigens displayed on tumors, and transfers the TILs expanded in vitro to the melanoma patients, which has shown clinical benefits in several studies conducted in past two decades [482, 483]. Recently, Phadke et al. tested combination of dabrafenib ± trametinib with pmel‐1 ACT for BRAF^mut^ melanoma in a preclinical trial. In this study, the antitumor activity was not only significant with the combination of dabrafenib, trametinib, and pmel‐1 ACT but also notable for dabrafenib or trametinib plus PD‐1 inhibitor in vitro. In vivo, dabrafenib plus pmel‐1 ACT had only modest tumor regression, while dabrafenib and trametinib combined with pmel‐1 ACT showed complete tumor regression. In contrast, dabrafenib alone increased immunosuppressive cell such as MDSCs, tumor‐associated macrophages (TAMs), and Tregs in tumors. The addition of trametinib to the combination of dabrafenib and pmel‐1 ACT did not increase TAMs and Tregs and further decreased their numbers. This could explain the significantly different antitumor effects observed in the triple combination therapy compared with dabrafenib plus pmel‐1 ACT [484]. While this triple combination therapy maybe a successful strategy for BRAF^mut^ melanoma, the efficacy for NRAS^mut^ is still unknown.

Lifileucel (LN‐144) is an autologous TILs therapy that uses TILs from patient's tumor‐tissue and being expanded ex vivo to produce polyclonal patient‐specific TILs (mix of CD8+ and CD4+ T cells) [485]. Results from a Phase II C‐144‐01 trial (NCT02360579) Cohort 2, which included melanoma patients with a mean of 3.3 prior therapies, showed that lifileucel had an ORR of 36% and a DCR of 80% [485]. The subsequent results from C‐144‐01 trial showed 153 patients (Cohort 2 + Cohort 4) received lifileucel with a median PFS of 4.1 months and a median OS of 13.9 months. More importantly, 41.7% patients with responses maintained for more than 18 months [486]. On February 16, 2024, US FDA approved lifileucel for advanced melanoma patients who had previously received ICIs.

There is another approach of ACT by identifying RAS‐mutant specific antigens for the treatment of NRAS^mut^ melanoma and have some breakthroughs in the treatment of NSCLC with KRAS mutation [487]. Polyclonal CD8+ TILs that specifically recognized the KRAS–G12D were identified from a metastatic colorectal cancer patient and reported by Tran et al. After the infusion of the expanded TILs, all seven pulmonary metastases achieved PR with a PFS of 9 months [488].

### 5.2 Rationale for Targeted‐Immunotherapy Combinations

#### 5.2.1 MEK Inhibitors–ICIs Sequence

There were several preclinical studies that demonstrated that MEK inhibitors can adjust TME of melanoma. On the one hand, inhibition of MEK can accumulate the infiltration of CD4+ and CD8+ T cell into the tumor, increase stem cell‐like memory T cells [489], increase expression of melanoma antigen and MHC I expression, which was associated with enhanced sensitivity to antigen‐specific T cells [490], promote the maturation of DCs [491], and decrease immunosuppressive cytokines (such as IL‐6 and IL‐10), which lead to reduced accumulation of MDSCs, TAMs, Tregs, and Bregs [492, 493]. Furthermore, MEK inhibitors improved anticancer T cell responses by impairing TCR‐mediated apoptosis of tumor antigen‐specific T cells [494, 495, 496, 497].

**FIGURE 3:**
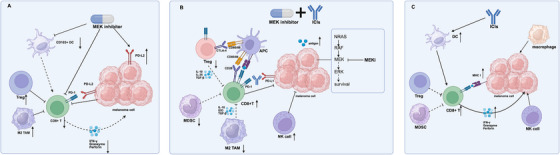
Mechanistic basis of the immunomodulatory effects of MEK inhibitor and their synergy with ICIs on the TME. (A) Mechanisms of MEKi and ICIs cross‐resistance (MEKi–ICIs sequence). (B) Mechanisms of the synergistic effect of MEKi and ICIs (MEKi combined with ICIs concurrently). (C) Mechanisms of ICIs–MEK inhibitors sequence. Dashed line: effect weaken. DC, dendritic cells; TAMs, tumor‐associated macrophages; MDSC, myeloid‐derived suppressor cells; Tregs, regulatory T cells; NK, natural killer cell.

On the other hand, inhibition of MEK leads to impair T cell proliferation (including naïve, CD4+, and CD8+ T cell) in vitro, reduce IFN‐γ production by T cells [498, 499], increase upregulating PD‐L2, which leads to the loss of T‐cell inflammation [494], and deleterious effects on antigen‐specific T cells [499]. After short‐term response, most melanoma cells that develop secondary resistance to MEK inhibitor underwent loss of antigen presentation [500], experienced epithelial‐to‐mesenchymal transition [501], and remodeled extracellular matrix, which was associated with increased the number of Tregs and M2‐TAMs and led to innate or adaptive resistance of ICIs [502]. Besides, reduced of CD103+ DCs also impeded the transportation of tumor antigens to draining lymph nodes and the activation of CD8+ T cell (Figure 3A) [503].

#### 5.2.2 ICIs and MEK Inhibitors Concurrently

Targeted therapy of BRAF^mut^ melanoma induces significant responses in the majority of patients, and the MEK inhibitors combined with RAF/ERK/PI3K inhibitors enhance clinical efficacy for NRAS^mut^ melanoma, while the DOR is often short‐term (NEMO trial: mPFS = 2.8 months; NCT05217303: mPFS = 4.2 months) [158, 169]. Conversely, treatment of melanoma with single agent of ICIs has lower ORR but can lead to more durable responses lasting for years [400, 403]. Therefore, it was suggested that combination of MEK inhibitors and PD‐1 inhibitors will significantly improve the outcome of NRAS mutation melanoma. Indeed, MEK inhibition robustly block naive CD8+ T cell, but truly increase the number of antigen‐specific CD8+ T cells within the tumor, and protect tumor‐infiltrating CD8+ T cells from apoptosis driven by chronic TCR stimulation while preserving their cytotoxic activity. Combining MEK inhibition with PD‐L1 inhibitor resulted in synergistic and durable tumor regression in tumor‐bearing mice compared with being only modestly effective in either single agent [497]. In preclinical studies, the combination approach has been associated with the released of antigens from tumor cells by targeted therapy, which could be more easily acknowledged by antigen‐specific T cells, decreased accumulation of TAMs and Tregs, as well as upregulated expression of PD‐L1 by improved IFNγ release, enhanced CD8+ T cell accumulation, and increased expression of MHC I and II (Figure 3B) [504].

#### 5.2.3 ICIs–MEK Inhibitors Sequence

The sequential schedules of ICIs and targeted therapy have been explored as a potential strategy to reduce levels of toxicity and improve durable antitumor responses. In a preclinical study reported by Phadke et al., the results showed that the sequence of immunotherapy and targeted therapy can modulate TME in NRAS‐mutated melanoma, leading to favorable changes such as increased infiltration of TIL and NK cells, and decreased presence of TAMs, MDCSs, and Tregs [505]. What is more, prior ICIs therapy increased the population of melanoma cells with overexpression of MHC I and melanoma antigens (Figure 3C) [505].

### 5.3 Clinical Experience and Challenges of Combinations

#### 5.3.1 MEK Inhibitors–ICIs Sequence in Clinical

Phase II SECOMBIT trial (NCT03235245) randomized 209 patients with metastatic BRAF^mut^ melanoma into Arm A (encorafenib plus binimetinib until PD followed by ipilimumab plus nivolumab), Arm B (ipilimumab plus nivolumab until PD followed by encorafenib plus binimetinib), and Arm C (encorafenib plus binimetinib for 8 weeks followed by ipilimumab plus nivolumab until PD and followed by encorafenib plus binimetinib) [506]. The primary endpoint 2‐year OS rate was 65% in Arm A, 73% in Arm B, and 69% in Arm C. The design of the DREAMseq trial (NCT02224781) is similar to that of the SECOMBIT trial, in which patients were randomly assigned to receive either ipilimumab/nivolumab (Arm A) or dabrafenib/trametinib (Arm B) with crossover to the alternate therapy upon PD [507]. In this study, the 2‐year OS rate was 71.8% in the Arm A and 51.5% in the Arm B (*p* = 0.01). Referring to the data from these two clinical trials, it supported the theory that acquired resistance to MEK inhibitors can induce cross‐resistance to ICIs, which is related to the immunosuppressive microenvironment [503].

#### 5.3.2 Concurrent Use of ICIs and MEK Inhibitors in Clinical

To maximize the clinical benefits of targeted‐therapy and immunotherapy, several studies have investigated concurrent combinations of MAPK inhibitors and ICIs in patients with advanced melanoma [508]; and some of them have examined the combinations of BRAF and MEK inhibitors with PD‐1/PD‐L1 inhibitors in patients with BRAF^mut^ melanoma [21, 22, 24, 509, 510, 511]. The Phase III IMspire150 study compared mPFS in vemurafenib and cobimetinib with or without atezolizumab for advanced BRAF mutation melanoma. The results showed that atezolizumab group had a significantly longer mPFS than control group (15.1 vs. 10.6 months, *p* = 0.025), and atezolizumab group was safe and tolerable [21]. Furthermore, second interim analysis of IMspire150 showed a trend toward prolonged mOS in the atezolizumab group compared with the control group (39.0 vs. 25.8 months, *p* = 0.14) [22]. Another Phase III clinical trial (COMBI‐I, NCT02967692) aimed to examine the effectiveness of dabrafenib and trametinib ± spartalizumab in patients with advanced BRAF^mut^ melanoma. The triple‐drugs therapy prolonged the mPFS compared with targeted therapy (16.2 vs. 12.0 months, *p* = 0.042; nonsignificant) [512]. According to the result from the KEYNOTE‐022 Phase II trial (NCT02130466), the median PFS of triple‐drugs (dabrafenib + trametinib + pembrolizumab) therapy and double‐drugs (dabrafenib + trametinib + placebo) for BRAF^mut^ melanoma were 16.9 and 10.3 months (*p* = 0.043); nevertheless, mPFS did not reach the planned benefit [24, 513]. Triple‐drugs therapy had a longer mPFS and mOS, demonstrating the synergistic effect of ICIs, MEK inhibitors and BRAF inhibitors.

The success of the combination of targeted therapy and immunotherapy make a promising for the treatment of NRAS mutation melanoma with MEK inhibitors plus ICIs. NCT02027961, a Phase I, open‐label, dose‐escalation and dose‐expansion study, tested the safety, tolerability, and preliminary efficacy of durvalumab (PD‐L1 inhibitor) combined with dabrafenib ± trametinib for advanced melanoma. In this study, they enrolled 68 patients including 11 patients with NRAS^mut^ melanoma, four of them received durvalumab concomitantly with trametinib, and seven of them received sequentially combined therapy (trametinib: Days 1–42; durvalumab: from Day 29) [508]. Three PRs were observed in 11 patients with NRAS‐mutant melanoma. The combination of naporafenib with spartalizumab (PD‐L1 inhibitor) was investigated in patients with advanced solid tumors harboring MAPK pathway alterations (including NRAS‐mutated melanoma). In this study, one complete response and 10 SDs were observed in the NRAS^mut^ melanoma (*n* = 21) [184]. Another randomized Phase III (IMspire170, NCT03273153) compared cobimetinib plus atezolizumab (PD‐L1 inhibitor) with single agent of pembrolizumab in patients with BRAF wild‐type melanoma (*n* = 71; 32% of combination group and 46.4% of pembrolizumab group with NRAS mutation). The combination of cobimetinib plus atezolizumab did not improve the mPFS compared with pembrolizumab in patients with advanced melanoma, and no differences were found in the prognosis and treatment efficacy of NF1 mutations, NRAS mutations (mPFS: 5.5 months of combination group and 5.7 months of pembrolizumab group), or other mutations in both groups [511]. For ORR, the combination group was lower than pembrolizumab group (26.6 vs. 36.3%). IMspire170 is the only Phase III trial that extensively enrolled NRAS mutation melanoma with ICIs plus MEK inhibitors. Meanwhile, IMspire170 results showed that the combination therapy failed to improve the efficacy and outcomes of NRAS‐mutated melanoma patients, and more TRAEs were observed in the combination therapy. In the future, larger‐scale clinical trials are needed to better understand the potential benefits and limitations of these combination therapies in NRAS/BRAF‐mutant melanoma. In the clinical trials, it is crucial to carefully monitor and manage TRAEs for the combination therapy. In IMspire170 study, the TRAEs above Grade 3 was 66.8%, and death of TRAEs was 3.2%, higher than the data of previous MEK inhibitors monotherapy (Table 4) [511].

**TABLE 4:** TRAEs of clinical trial for NRAS mutation melanoma.

Trial	Phase	Patients	Therapy	TRAE (≥G3) %	TRAE (≥G3)	TRAEs interruption	TRAEs death
NCT01320085	II	30 NRAS^mut^	MEK162	43.3%	Increase blood CK 23%; diarrhea 7%; oedema 7%	13%	0
NCT01763164	III	269 NRAS^mut^	MEK162	65.8%	Increased blood CK 19%, hypertension 7%, anemia 2%	25%	2.2%
NCT01763164	III	114 NRAS^mut^	Dacarbazine	44.7%	Anemia 5%; neutropenia 4%; asthenia 4%	8%	0.8%
NCT03932253	Ia	33 NRAS^mut^	FCN‐159	15.2%	Anemia 3%; peripheral oedema 3%	15.2%	3%
NCT03973151	I	42 NRAS^mut^	HL‐085	38.1%	Increased CK 14.2%; asthenia 7.1%, peripheral edema 4.8%	7.1%	0
NCT00982865	I	20/89 NRAS^mut^	Pimasertib	8.9%	Skin event 4.5%, ocular event 2.2%, diarrhea 2.2%	NM	0
NCT01693068	II	130 NRAS^mut^	Pimasertib	85%	Increased CK 34%, hypertension 9%, dermatitis acneiform 7%	47%	1%
NCT00338130	II	10/104 NRAS^mut^	Selumetinib	57.6%	Dermatitis acneiform 12.1%, diarrhea 4%, nausea 3%	10.1%	3%
NCT01425008	I	17/19 NRAS^mut^	Tovorafenib	47%	Anemia 11%, rash maculo‐papular 5%, dyspnea 5%	21%	0
NCT01941927	II	10/20 NRAS^mut^	Trametinib + GSK2141795	30%	Rash 20%, dehydration 5%, diarrhea 5%	NM	0
NCT01781572	II	41 NRAS^mut^	Ribociclib + binimetinib	92.7%	Increased CK 24.4%, increased AST 22%, increased ALT 24.4%	26.8%	7.3%
NCT01781572	Ib	29 NRAS^mut^ (28 day schedule)	Ribociclib + binimetinib	89.7%	Increased CK 17.2%, increased AST 13.8%, increased ALT 13.8%	24.7%	13.8%
DOC‐MEK	II	20/41 NRAS^mut^	Selumetinib + docetaxel	78.9%	fatigue 21%, febrile neutropenia 21%, rash 13%	NM	NM
NCT02110355	I	31 included NRAS^mut^	AMG 232 + DT/T	29%	Nausea 13%, diarrhea 10%, vomiting 10%, pulmonary embolism 6.5%	23%	3.2%
NCT02138292	Ib	6/20 NRAS^mut^	Digoxin + trametinib	40%	Diarrhea 20%, fatigue 5%, confusion 5%	0	0
NCT00121680	Ib	6/32 NRAS^mut^	Lenvatinib + temozolomide	40.6%	Asthenia 12.5%, hypertension 9.4%, fatigue 6.3%	0	0
PMID26169970	I	8/26 NRAS^mut^	Lenvatinib	61.5%	Hypertension 23.1%, fatigue 11.5%, proteinuria 11.5%	0	0
NCT02027961	I	7/22 NRAS^mut^	Durvalumab + trametinib	72.7%	Increased CK 13.6%, rash 9.1%, diarrhea 4.5%	50%	4.5%
NCT02607813	I	21 NRAS^mut^	Naporafenib + spartalizumab	38.1%	Rash 14.3%	NM	NM
NCT03273153	III	71/222 NRAS^mut^	Cobimetinib + atezolizumab	66.8%	Increased CK 10%, diarrhea 7.7%, rash 6.8%	11.8%	3.2%
NCT03273153	III	104/224NRAS^mut^	Pembrolizumab	33.3%	Hypertension 3.7%, pulmonary embolism 2.3%, diarrhea 1.9%	0.9%	2.3%
NCT01676649	II	2/30 NRAS^mut^	Ipilimumab + carboplatin/paclitaxel	70%	Neutropenia 17%, diarrhea 10%, thrombocytopenia 10%	10%	0

*Data source*: Clinical data obtained from https://clinicaltrials.gov.

Abbreviations: CK, creatine phosphokinase; D, dabrafenib; T, trametinib; NM, not mention.

The combination of targeted therapy and ICIs (especially ipilimumab) commonly occurs severe off‐target effects and leads to the suspension of the treatment [514].

#### 5.3.3 ICIs–MEK Inhibitors Sequence in Clinical

At least two prospective clinical studies observed the sequence of ICIs and MEK inhibitor improved the efficacy and prognosis for NRAS^mut^ melanoma patients. In the subgroup analysis of NEMO study, patients who were pretreated with immunotherapy benefit more from binimetinib than dacarbazine (mPFS: 5.5 vs. 1.6 months; ORR: 16 vs. 4%) [158]. In patients of the NCT05217303 study who received previous ICIs therapy, the ORR of tunlametinib was higher than patients without previous ICIs therapy (40.6 vs. 25.8%) [169]. Two ongoing clinical trials are further investigating the sequence of targeted therapy and ICIs therapy. NCT03149029 investigating the sequence of BRAF–MEK inhibitor (trametinib with dabrafenib) and anti‐PD‐1 (pembrolizumab) in BRAF^mut^ melanoma. Another ongoing Phase III trial, NCT02224781, aimed to compare 2 years over survival of ipilimumab + nivolumab with subsequent dabrafenib plus trametinib and dabrafenib + trametinib with subsequent ipilimumab plus nivolumab in advanced BRAF^mut^ melanoma.

## 6 Conclusion and Future Perspectives

Targeting the MAPK signaling pathway has significantly improved the treatment of metastatic BRAF/NRAS mutation melanoma. MEK inhibitors combined with MAPK pathway inhibitors or PI3K–AKT–mTOR inhibitors have shown synergistic effect in several preclinical studies, while their validation in clinical trials is lacking. In the not‐so‐far future, researchers should focus on the results of ongoing clinical trials of MEK inhibitors combination therapy, and we summarized the ongoing clinical trials for NRAS^mut^ melanoma.

On the other hand, acquired resistance of MEK inhibitors and BRAF inhibitors remains a challenge in the treatment of NRAS/BRAF‐mutant melanoma. Therefore, exploring the intrinsic and acquired resistance mechanisms to MEK inhibitors or MEK inhibitors combination therapy is utmost importance in the future. Moreover, broad‐spectrum RAS inhibitors provide therapeutic benefit for RAS^mut^ tumors, and the clinical trials are ongoing. More recently, the researchers observed a pocket near Switch II of NRAS Q61K mutant, which may influence the development of NRAS inhibitors [515].

Immunotherapy is another strategy for the treatment of NRAS^mut^ and BRAF^mut^ melanoma. ICIs such as anti‐PD‐1, anti‐PD‐L1, and anti‐LAG3 improved the outcome of advanced melanoma in Phase III trials. Other ICIs such as anti‐TIGIT, anti‐TIM3, anti‐CD137, and bispecific antibody (cadonilimab), and immunomodulators such as IDO inhibitors and TLRs inhibitors are testing for advanced melanoma [487, 516]. Personalized tumor neo‐antigens vaccines (such as BNT111) target the undruggable mutation, when combined with ICIs maybe transforming the way of NRAS^mut^ melanoma treatment [517]. Meanwhile, breaking the immunosuppressive TME of advanced melanoma is a worth exploring strategy to improve the efficacy of ICIs and overcome the resistance of ICIs.

For now, the sequential schedules of ICIs and MEK inhibitors maybe the optimal solution for BRAF^mut^/NRAS^mut^ melanoma. In order to further improve the efficacy and prognosis of BRAF^mut^/NRAS^mut^ melanoma patients, the combination of MEK inhibitors and ICIs will be a hot topic for some time to come. Based on the clinical trials of single agent of MEK inhibitors or monotherapy of ICIs, most of them toxicity is controllable. And, careful attention should be given to TRAEs when combining these therapies.

## Author Contributions

Y.F., J.L., and Z.M. contributed to the manuscript writing and figure preparation. Y.F., B.W., Y.D., and Y.J. designed the work. Y.D. and Y.J. supervised the work. All authors have read and approved the final manuscript.

## Ethics Statement

The authors have nothing to report.

## Conflicts of Interest

The authors declare no conflicts of interest.

## Data Availability

The authors have nothing to report.
